# The ASH1 HOMOLOG 2 (ASHH2) Histone H3 Methyltransferase Is Required for Ovule and Anther Development in *Arabidopsis*

**DOI:** 10.1371/journal.pone.0007817

**Published:** 2009-11-12

**Authors:** Paul E. Grini, Tage Thorstensen, Vibeke Alm, Gema Vizcay-Barrena, Susanne S. Windju, Tommy S. Jørstad, Zoe A. Wilson, Reidunn B. Aalen

**Affiliations:** 1 Department of Molecular Biosciences, University of Oslo, Oslo, Norway; 2 School of Biosciences, University of Nottingham, Nottingham, United Kingdom; 3 Department of Biology, Norwegian University of Sciences and Technology, Trondheim, Norway; Umeå Plant Science Centre, Sweden

## Abstract

**Background:**

SET-domain proteins are histone lysine (K) methyltransferases (HMTase) implicated in defining transcriptionally permissive or repressive chromatin. The *Arabidopsis* ASH1 HOMOLOG 2 (ASHH2) protein (also called SDG8, EFS and CCR1) has been suggested to methylate H3K4 and/or H3K36 and is similar to *Drosophila* ASH1, a positive maintainer of gene expression, and yeast Set2, a H3K36 HMTase. Mutation of the *ASHH2* gene has pleiotropic developmental effects. Here we focus on the role of *ASHH2* in plant reproduction.

**Methodology/Principal Findings:**

A slightly reduced transmission of the *ashh2* allele in reciprocal crosses implied involvement in gametogenesis or gamete function. However, the main requirement of *ASHH2* is sporophytic. On the female side, close to 80% of mature ovules lack embryo sac. On the male side, anthers frequently develop without pollen sacs or with specific defects in the tapetum layer, resulting in reduction in the number of functional pollen per anther by up to ∼90%. In consistence with the phenotypic findings, an *ASHH2* promoter-reporter gene was expressed at the site of megaspore mother cell formation as well as tapetum layers and pollen. *ashh2* mutations also result in homeotic changes in floral organ identity. Transcriptional profiling identified more than 300 up-regulated and 600 down-regulated genes in *ashh2* mutant inflorescences, whereof the latter included genes involved in determination of floral organ identity, embryo sac and anther/pollen development. This was confirmed by real-time PCR. In the chromatin of such genes (*AP1*, *AtDMC1* and *MYB99*) we observed a reduction of H3K36 trimethylation (me3), but not H3K4me3 or H3K36me2.

**Conclusions/Significance:**

The severe distortion of reproductive organ development in *ashh2* mutants, argues that ASHH2 is required for the correct expression of genes essential to reproductive development. The reduction in the *ashh2* mutant of H3K36me3 on down-regulated genes relevant to the observed defects, implicates ASHH2 in regulation of gene expression via H3K36 trimethylation in chromatin of *Arabidopsis* inflorescences.

## Introduction

Active transcription of eukaryotic genes is dependent on a permissive chromatin structure that allows transcription factors access to promoter and enhancer regions. The accessibility of chromatin depends on the position and packaging of the nucleosomes and relies upon enzymes that modify residues on the protruding histone tails, or recognize such modifications. A number of histone tail modifications have been identified including acetylation, phosphorylation, ubiquitination, and methylation [1]. While acetylation of lysine (K) residues in general is associated with transcriptional activity, methylation of lysines can be associated with maintenance of an active or a repressed state depending on the position of the modified residues. In general, euchromatin contains elevated levels of histone H3 lysine methylation at positions 4, 36 and 79 as well as hyperacetylation of histone H4 [2].

Enzymes that can add methyl groups to lysine residues on histone tails can be grouped in evolutionarily conserved classes named after the *Drosopohila* proteins SU(VAR)3–9, E(Z), TRITHORAX (TRX) and ASH1, that all have a similar 130 amino acid (aa) long SET domain [3], [4]. Proteins belonging to the ASH1 class have properties similar to TRX in maintenance of transcription during development. These four classes have also been identified in *Arabidopsis thaliana* [5]. In the *Arabidopsis* ASH1 class there are four ASH1 HOMOLOGs (ASHH) and three ASH1 RELATED (ASHR) members [5]. *ASHH2* (At1g77300, called *SET DOMAIN GROUP 8* (*SDG8*) in the Plant Chromatin Database http://www.chromdb.org) was the first *Arabidopsis* gene of the ASH1 class to be ascribed a biological function, namely in controlling flowering time. This gene is also named *EFS* as it was identified as the one affected in the mutant *early flowering in short days (efs)* in the Landsberg *erecta* (L*er*) ecotype, and mutations also result in earlier flowering in the Colombia (Col) ecotype (Table 1) [6], [7]. The early flowering phenotype correlated to transcriptional repression of the key regulator of flowering time, *FLOWERING LOCUS C* (*FLC*) [6]–[8]. *FLC*-centered studies have been crucial for the elucidation of the molecular mechanisms of epigenetic gene regulation [9]. However, several genes affecting *FLC*, including *ASHH2*, *ATX1*, *ELF7* and *ELF8*, display additional mutant phenotypes, in plant size, flower morphology and/or fertility [8], [10], [11]. Recently, *ASHH2* has also been shown to be involved in regulating shoot branching and carotenoid composition (Table 1) [12], [13].

**Table 1:** Alleles of *ashh2*.

Allele	Ecotype	Mutation	Described phenotype	Affected genes	Reference
*efs-1, efs-2*	L*er*	γ irradiation	Early flowering in short days		Soppe et al., 1999
*efs-3, efs-5, esf-6, efs-7*	*FRI*-Col	fast-neutron radiation	Early flowering	*FLC, MAF1, MAF2*	Kim et al., 2005
*esf-4, esf-8*	*FRI-*Col	T-DNA insertions	Early flowering		
*esf-9, efs-10, esf-11*	Ws	T-DNA insertions	Early flowering in short days		
*sdg8-1, sdg8-2, sdg8-3*	Col	T-DNA insertions	Early flowering	*FLC*	Zhao et al., 2005
*sdg8-1, sdg8-2,*	Col	T-DNA insertions	Early flowering	*FLC, MAF1, MAF2, MAF3, MAF4, MAF5*	Xu et al., 2008
*sdg8-2, sdg8-4*	Col	T-DNA insertions	Shoot branching	*SPS, UGT74E2*	Dong et al., 2008
*ccr1-1, ccr1-2, ccr1-4, ccr1-5, ccr1-6, ccr1-7*	Col	EMS	Carotenoid composition and shoot branching	*CRTISO*	Cazzonelli et al., 2009
*ashh2-1/sdg8-1* a, *ashh2-2/sdg8-2* a, *ashh2-5, ashh2-6*	Col	T-DNA insertions	Homeotic changes in floral organs; distorted development of reproductive organs	*AtDMC1, AP1, MYB99*	The present work

a The *ashh2-1* and *assh2-2* alleles are identical to *sdg8-1* and *sdg8-2*.

These additional phenotypes are worth investigations as they suggest epigenetic gene regulation in other developmental processes. In the present paper we have therefore examined the *ashh2* mutant phenotype with respect to causes of severely reduced fertility, which was already noted by [8]. Serious defects in both male and female reproductive organ development are observed. In a microarray experiment comparing gene expression in *ashh2* and wild type (wt) inflorescences, followed by quantitative RT-PCR confirmation, we have identified genes associated with the mutant phenotype with significantly reduced expression levels.

*In vitro* ASHH2 shows histone H3 methyltransferase (HMTase) activity on oligonucleosomes and core histones [12], [14]; but activity on unmodified recombinant histone tails has not been demonstrated, suggesting that the protein need some premodification or cofactor to function. Thus the exact specificity is not known, but Chromatin Immuno Precipitation (ChIP) at *FLC* and other loci in *ashh2/sdg8/efs/ccr1* mutants compared to wt has suggested that ASHH2 is a H3K4 trimethyltransferase [6], [13] and/or a H3K36 di- and trimethyltransferase [7], [12], [14]. We have used antibodies against these marks in our ChIP experiments on chromatin from inflorescences, and demonstrate significant reduction of H3K36me3 methylation, but not H3K4me3 or H3K36me2 on down-regulated genes associated with the mutant *ashh2* phenotypes. This suggests that ASHH2 controls development of *Arabidopsis* reproductive organs via H3K36 trimethylation.

## Results

### Mutation in *ASHH2* results in a pleiotropic phenotype

**Figure 1:**
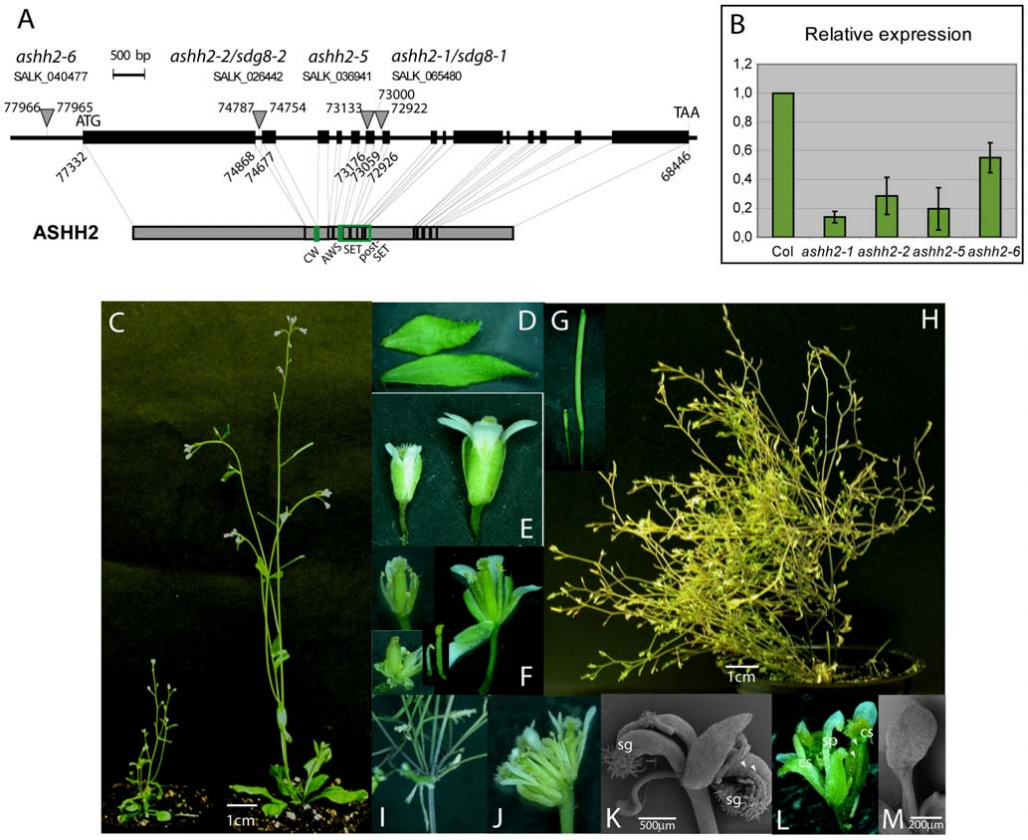
Alleles of *ashh2* and the pleiotropic phenotype of *ashh2* mutant plants. (A) Position of the T-DNA insertions of different SALK lines as indicated. The 15 exons of *ASHH2* are shown as black boxes and the introns as thin lines. Numbers refer to base pairs (bp) in the *Arabidopsis* genomic BAC clone T14N5 (acc.no. AC004260) and indicate insertion sites of the T-DNAs, start and stop codons and selected exon-intron borders. The positions of primers used for real-time PCR are show in green above exons 10 and 11. Both the right and the left junction between the *ASHH2* gene and the insertions were cloned and sequenced for all alleles except *ashh2-5* which is likely to have a complex insertion. Genomic deletions of 33 and 8 bp where found at the insertion sites of *ashh2-2* and *ashh2-1*, respectively. The lower panel shows the ASHH2 protein and the positions of known domains. (B) Relative expression level of *ASHH2* in *ashh2* mutant plants. Real-time PCR on cDNA from inflorescences of plants homozygous for the indicated alleles as compared to the level in wt inflorescences (set to 1). Standard deviations are shown. (C) Dwarf phenotype of *ashh2-1* with several inflorescences (left) compared to wt plant with main inflorescence and a shorter auxiliary shoot (right). (D) Small *ashh2-1* cauline leaf (top) and wt cauline leaf (bottom). (E) Small *ashh2-1* flower (left) compared to wt flower (right). (F) *ashh2-1* flowers with mild (top left) and more severe (bottom left) distorted stamens and carpels compared to wt flowers (right). Some sepals and petals have been removed to display the inner whorl organs. *ashh2-1* stamens are often shorter than wt stamens (bottom middle). (G) Siliques of *ashh2-1* plant (left) and wt plant (right). (H) *ashh2-1* plant 100 days after sowing. (I) Highly branched internode of *ashh2-1* plant 100 days after sowing. (J) Composite flower of *ashh2-1* plant 100 days after sowing. (K) Scanning electron micrograph (SEM) of *ashh2-1* flower with sepals and carpeloid organs with vestigial ovules (ov) and stigmatic papillae (sg). (L) *ashh2-1* flower with carpeloid sepals (cs) and stamenoid petals (sp). (M) SEM of stamenoid petal of *ashh2-1* flower.

Four *ashh2* alleles were analyzed; with T-DNAs inserted in the promoter 643 bp upstream of the translation start (*ashh2-6*), in the first intron (*ashh2-2/sdg8-2*), in the sixth exon (*ashh2-5*) and the sixth intron/seventh exon (*ashh2-1/sdg8-1*), respectively (Figure 1A, Table 1). In the latter three alleles the *ASHH2* expression levels are strongly reduced compared to wt (Figure 1B). These alleles are not expected to produce functional proteins, as the T-DNAs are positioned upstream of or in the region encoding the SET domain, suggesting that they are null alleles. Plants homozygous for *ashh2-1*, *ashh2-2* and *ashh2-5* show a characteristic dwarf and bushy phenotype (Figures 1C and 1H, and Figure S1). This phenotype was not found in *ashh2-6* plants, the only allele that can be expected to produce a normal transcript and for which the expression level in inflorescences was only reduced to 55% (Figure 1B and Figure S1). We have mainly focused on the phenotypes found to be common to all alleles. All *ashh2* organs were smaller than wt organs (Figures 1D and 1E). Stamens were shorter with thinner filaments that often were bent just beneath the anther, while the carpels were shorter and thicker compared to wt (Figure 1F). *ashh2* siliques developed poorly (Figure 1G), and flowering and growth continued for at least 100 days (Figure 1H). These plants had highly branched internodes (Figure 1I) and composite or clustered flowers without pedicels (Figure 1J).

Inflorescences with flowers exhibiting homeotic transformations were consistently observed in all alleles inspected, including the *efs1-1* allele in L*er* background (Table S1). The transformed flowers mainly displayed sepals and carpeloid organs (Figure 1K) or carpeloid sepals and stamen-like petals (Figures 1L and 1M). Homeotic changes with sepal and petal organs only, were never encountered (*n = *83, Table S1).

### *ashh2* transmission is affected through both male and female gametes

**Figure 2:**
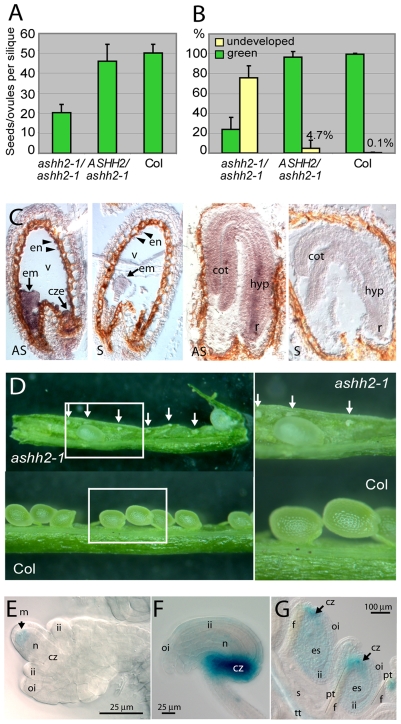
Ovule development and seed set in *ashh2* mutant plants, and *pASHH2:GUS* expression during ovule and seed development. (A) Total number of seeds/ovules per silique in selfed wt (*n*_siliques_ = 12), homozygous (*n*_siliques_ = 125) and heterozygous *ashh2-1* plants (*n*_siliques_ = 26). (B) Percentage of developing green seeds and undeveloped ovules per silique in selfed wt (*n*_siliques_ = 12), homozygous (*n*_siliques_ = 125) and heterozygous *ashh2* plants (*n*_siliques_ = 26) prior to seed desiccation. (C) *In situ* hybridization on longitudinal sections of seeds at the heart stage (left) and the walking stick stage (right) of embryo development with *ASHH2* antisense probe (AS) and control sense probe (S). Expression was seen in the whole heart stage embryo (em), the chalazal endosperm (cze) as well as the endosperm nodules (en) aligning the embryo sac around the vacuole (v). At the walking stick stage expression was seen both in the cotyledons (cot), hypocotyl (hyp) and root tip (r). (D) Full-length *ashh2-1* mutant silique compared to a segment of a wt siliques, with enlarged sections to the right. Arrows point to undeveloped ovules. (E) *pASHH2:GUS* expression in the nucellus of the developing ovule. The GUS signal is located in the L2 layer where the megaspore (m) forms. Nucellar tissue (n), chalazal tissue (cz), inner integuments (ii), outer integuments (oi). (F) *pASHH2:GUS* expression in the ovule with mature embryo sac. Strong expression is found in maternal chalazal proliferating tissue (cz). Weaker expression was observed in the nucellus and inner (ii) and outer integuments (oi). (G) *pASHH2:GUS* expression in maternal tissue strongly in the chalazal tissues (cz) and weaker in the inner integuments (ii) and even weaken in the outer integument (oi) after fertilization. The chalazal region (cz) is indicated by an arrow and pollen tubes (pt) that have grown along the funiculus (f) with thin lines. The septum (s) with its transmitting tract (tt) is indicated.

Mutant plants of all alleles had short siliques. In *ashh2-1* siliques the ovule number was reduced by 80% compared to wt (Figure 2A), and less than a quarter of the ovules developed into mature seeds (Figures 2B and 2D). Reduced seed setting was also observed after hand self-pollination of the mutant. *In situ* hybridization demonstrated expression of *ASHH2* in the endosperm as well as in the developing embryo (Figure 2C). If the reduced seed set was due to developmental defects in homozygous *ashh2* fertilization products, one would have expected 25% aborted seeds when self-fertilizing heterozygous plants. However, *ASHH2* appears to be redundant both for embryo and endosperm development as no deviation from wt in number of ovules/seeds or phenotype could be observed during embryogenesis in siliques of selfed heterozygous *ASHH2*/*ashh2-1* plants (Figure 2B).

If the reduced seed set was due to a defect in *ashh2* haploid gametophytes, one would expect reduced transmission of the mutant allele. Therefore we genotyped the progeny resulting from self-fertilization of heterozygous plants. Self-fertilization of *ASHH2/ashh2-1* plants resulted in only 8% homozygous *ashh2-1*/*ashh2-1* mutant progeny and 37% wt plants, instead of the expected 25% (*n* = 62, Table 2). A similar frequency of homozygous plants resulted from selfing of heterozygous *ASHH2*/*ashh2-2*, *ASHH2*/*ashh2-5* and *ASHH2*/*ashh2-6* plants (10.7%, *n* = 140; 8.5%, *n* = 71; 17%, *n* = 94, respectively). As no seed abortion could be observed, this suggested that male and/or female transmission was affected. This was tested with reciprocal crosses of *ASHH2*/*ashh2-1* heterozygous plants with wt plants, which revealed that both female and male transmission was reduced from the expected 50% to 39% and 37%, respectively (Table 2). The transmission efficiency (Table 2) for the male and female mutant alleles would result in an expected frequency of *ashh2* homozygous progeny of 9%, i.e. close to the observed 8%. The expected number of male and female gametophytes in *ASHH2/ashh2* plants defective in transmission of mutant gametes would be about 20% (Table 2) [15]. Since no obvious defects in pollen or embryo sac development could be observed in heterozygous lines, this suggests a role for ASHH2 in gametophyte function, e.g. pollen tube growth or attraction.

**Table 2:** Segregation of *ASHH2/ashh2* in reciprocal crosses with wt.

Female X Male	*ASHH2/ASHH2*	*ASHH2/ashh2*	*ashh2/assh2*	N	T^a^	TE^b^	Δ^c^	EXP homo^d^
***ASHH2/ashh2-1*** ** selfed**	37%	55%	8%	62	N.D.	N.D.	N.D.	9%
***ASHH2/ashh2-1*** ** x Col**	61%	39%	0%	129	0.78	0.58	21%	N.D.
**Col x ** ***ASHH2/ashh2-*** **1**	63%	37%	0%	142	0.74	0.59	20%	N.D.

a Transmission of mutant allele (T) was calculated as T(observed)/T(expected).

b Transmission efficiency (TE) calculated as observed number of transmitted mutant alleles (TEobs)/expected number of transmitted mutant alleles (TEexp). TEexp for the mutant allele correspond to the observed number of WT alleles transmitted in the reciprocal cross (Howden et al., 1998).

c Expected frequency of defective male or female gametophytes in a *ASHH2/ashh2* plant. Δ = 0.5 (1-TE)×100%.

d Expected frequency of homozygous plant calculated from TE(obs)female and TE(obs)male.

The frequency of affected female or male gametophytes alone could not account for the severe reduction in seed set observed in homozygous *ashh2* plants (Figures 2A, 2B and 2D). Thus this suggests that the main requirement of ASHH2 is in the sporophyte for proper development or function of ovules and anthers.

### *ashh2* mutants are defective in ovule and embryo sac development

**Figure 3:**
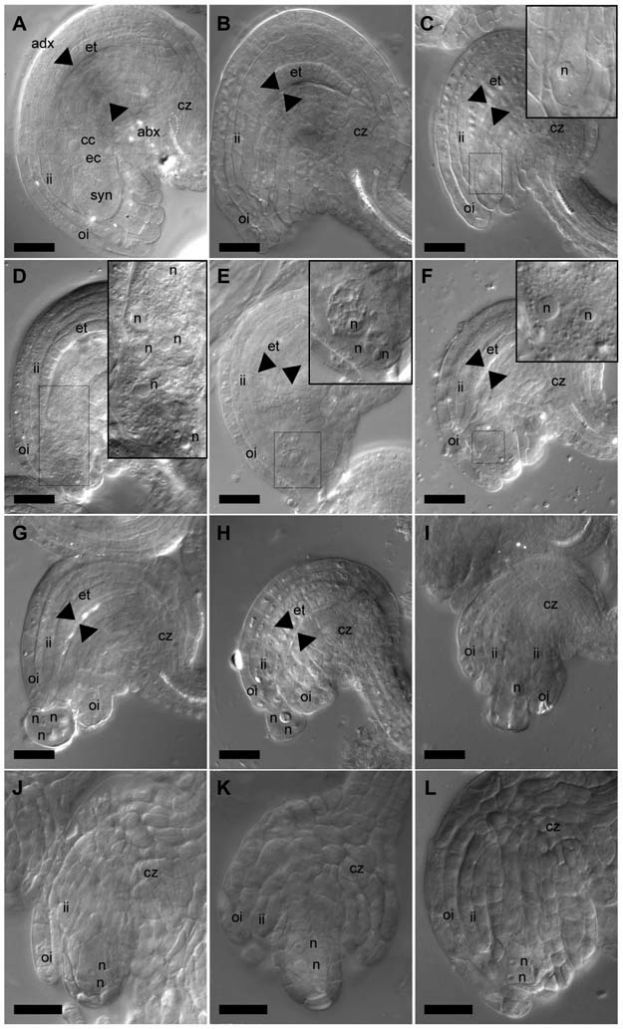
Ovule and embryo sac development in wt and *ashh2-1*. Differential interference contrast (DIC) micrographs of (A) wt and (B–L) *ashh2-1* ovules at one day post anthesis (A–C), anthesis (D–F), day before anthesis (G–I) and at the time point of the first syncytial nuclear division (J–L). Scale bar, 25 µm. (A) Wt mature ovule harboring embryo sac with (arrows) egg cell (ec), central cell (cc) and synergid cells (syn, only one synergid cell is in the focal plane). The embryo sac is surrounded by maternal chalazal (cz) tissue and endothelium layer (et), and further surrounded by two more layers of inner integuments (ii) and two layers of outer integuments (oi). Arrowheads mark the border between the embryo sac and the adaxial (adx) endothelium and the abaxial (abx) chalazal tissue. (B) *ashh2-1* ovule without embryo sac between endothelium and the adaxial chalazal tissue (arrowheads). Ovule morphology appears fairly normal with two outer integument layers (oi) and three inner integuments (ii and et). The chalazal tissue in the center of the ovule appears to have proliferated more than in wt. (C) *ashh2-1* ovule from same silique as (B) with a single mono-nucleate cell (boxed). Inset in the upper right corner shows magnification of boxed area (n, nucleus). The outer integuments engage both inner integuments and nucellus. (D) *ashh2-1* syncytial embryo sac with eight nuclei. Inset in the upper right corner shows magnification of boxed area (n, nucleus). Two nuclei are not in the focal plane. (E) Proliferated *ashh2-1* syncytial embryo sac. Inset in the upper right corner is a merge of two focal planes and shows magnification of the boxed area (n, nucleus). The basally located nuclei appear to degenerate. Note that the micropylar end of the embryo sac is not covered by integuments in both (E) and (F). (F) *ashh2-1* embryo sac. Inset in the upper right corner shows magnification of the boxed area show two nuclear structures reminiscent of egg and central cell nuclei (n, nucleus). (G–H) *ashh2-1* ovules with embryo sac-like structure (arrow) budding off the micropylar end of the ovule. The integuments do not encase the structure. (I) *ashh2-1* ovule arrested or delayed in development. The inner integuments have developed longer than the outer integuments. (J–L) *ashh2-1* ovules with two nucleate syncytial embryo sacs. No vacuole could be observed between the nuclei (n, nucleus).

In wt plants, one megaspore resulting from meiosis of the megaspore mother cell (MMC) develops into the embryo sac comprised of the egg cell, two synergid cells, three antipodal cells, and a dihaploid central cell (Figure 3A). Inner- and outer integuments elongate in the proximal-distal axis and encase the nucellar tissue.

One day post anthesis (flower stage 12) when wt ovules harbor a mature embryo sac (Figure 3A), no apparent embryo sac, or in a few cases an embryo sac consisting of a single-nucleated cell, was found in mutant ovules (Figures 3B and 3C; 79%, *n* = 141). The overall ovule and integument morphology was close to wt in this class, however, an overproliferation of the abaxial chalazal part of the ovule was often observed (Figure 3B). In slightly earlier stages, a small fraction of ovules could be found where up to three rounds of nuclear proliferation had occurred in the embryo sac (Figures 3D to 3F; 9%, *n* = 141, in corresponding wt stages 95%, n = 111). In most cases, these syncytial embryo sacs were not encased completely by the outer integuments (Figures 3E and 3F). The observed proliferating nuclei were in most cases undifferentiated (Figure 3D) or appeared to start to degenerate (Figure 3E), but nuclear structures reminiscent of egg and central cell nuclei were also observed (Figure 3F). In some cases (10%, *n* = 141) the embryo sac-like structure was not surrounded by the integuments and protruded out of the micropyle as a “bag” of nuclei (Figures 3G and 3H). The nuclei in some cases appeared degenerated (Figure 3G) or were of different sizes (Figure 3H). In ovules that aborted in early stages both outer and inner integument elongation appeared to be delayed (Figure 3I).

In order to investigate whether embryo sacs develop and thereafter are expelled from the ovule, thus resulting in an apparent lack of the female gametophyte, or if most embryo sacs abort and degenerate at early stages of development, we analyzed young stages of embryo sac development. We found that the majority of the embryo sacs inspected (66%, n = 62) were degenerated and no clear nuclear morphology could be seen. In 18% of the embryo sacs a prominent single nuclei could be found (similar to Figure 3C, n = 62). However, 16% of the embryo sacs had completed the first nuclear division and harbored two syncytial nuclei (Figure 3J–L, n = 62). Our findings thus favor a scenario where most *ashh2* embryo sacs arrest and degenerate slightly after or before the first syncytial division. The number of embryo sacs that have made the first nuclear division is in good accordance with the number of protruding embryo sacs found at later stages, suggesting that if the first division is made further divisions can follow, but that most of these syncytial embryo sacs will be expelled, resulting in an apparent lack of the female gametophyte.

Taken together the observed defect in *ashh2* ovules appears to be a failure to progress into female gametophyte or embryo sac development after meiosis. Based on the genetics of the mutant lines, a sporophytic requirement of ASHH2 is suggested and thus the primary defect may relay on a failure in meiosis or MMC initiation. In support of this hypothesis, a construct with the *ASHH2* promoter coupled to the *GUS* reporter gene, was expressed in the subepidermal L2 cell layer of the nucellus at the position of MMC initiation (Figure 2E) [16]. The observed phenotype could alternatively be effected by missing support from the maternal ovule integuments. However, during later stages of the mature embryo sac, *pASHH2:GUS* expression was most prominent in maternal chalazal proliferating tissue and only weakly expressed in the integuments (Figures 2F to 2G).

### *ashh2* plants have reduced numbers of pollen

**Figure 4:**
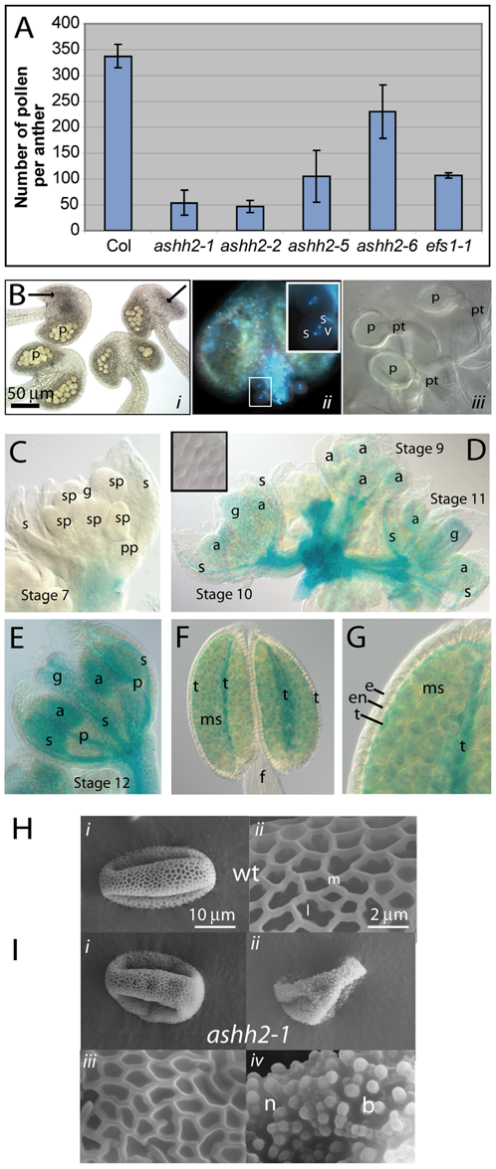
Pollen development in *ashh2* and *pASHH2:GUS* expression in anther development. (A) Average number of pollen per anther (*n_wt_* = 4, n*_ashh2_*>8) in wt and *ashh2* mutant lines. Standard deviations are shown. (B) Light- and fluorescent micrographs of *ashh2-1* anthers and germinating pollen tubes. (*i*) Four long stamina from *ashh2-1* from a flower just before abscission. All anthers are delayed in dehiscence. Note different numbers of pollen (p) in each locule and absence of normal locule development (arrows). (*ii*) Mature *ashh2-1* anther with released pollen in the trinucleate stage (boxed area). Inset is a magnification of the boxed area, (s) sperm cell, (v) vegetative cell. (*iii*) Pollen tube germination on the papillae in *ashh2-1*. (p) pollen, (pt) pollen tube. (C) Stage 7 flower of *pASHH2:GUS* plant. No GUS expression was seen in the developing floral organs but only in the pedicel. s – sepal; g – gynoecium; pp – petal primordium; sp – stamen primordium. (D) Stage 9 to 11 flowers showing increasing GUS expression post meiosis. The boxed insert shows that no GUS expression was detected in tetrads. s – sepal; g – gynoecium; a – anther. (E) Stage 12 flower with mature anthers with GUS expression in anthers (a), as well as the gynoecium (g) and mid veins of petals (p) and sepals (s). (F) Anther of stage 12 flower showing GUS expression both in the tapetal cell layer (t) and the mature microspores (ms), but not in the anther filaments (f). (G) Detail of GUS expression in floral stage 12 anther demonstrate specific expression in tapetum (t) and microspores (ms), but not in the surrounding endothecium (en) and epidermis (e) cell layers. (H) Scanning electron micrographs of wt pollen (*i*) with a sexine layer (*ii*) with regular ridges (muri – m) and spaces (lumina – l). (I) Scanning electron micrographs of *ashh2-1* pollen grains (*i, ii*) and exine layers with filled muris (*iii*), and in severe cases visible nexine layer (n) and bacula (b) due to absent tectum layer (*iv*).

Due to the low expected frequency of affected pollen grains in heterozygous plants (Table 2) male semi-sterility was studied in homozygous mutants. All mutant alleles had reduced amounts of pollen (Figure 4A); anthers displayed a large variation in the number of pollen in each pollen sac (locule) with frequent empty or undeveloped locules (Figure 4B, Figure S2), e.g. ∼1.6 locules were seen per *ashh2-1* anther (total 49 locules in 31 anthers). This suggests that the early stages of archesporial division have been affected, resulting in an absence of the parental cell layers generating the sporocytes.

As the reduced number of pollen grains in *ashh2* could also be due to defects in male meiosis, we inspected meiosis in *ashh2-1*, *ashh2-2* and *ashh2-5* lines using a whole-mount DAPI/Aniline Blue staining procedure. Tetrad number from each anther was very variable and generally lower in the mutant, however, examination of stages from the initial division of the pollen mother cells (PMC) up to early progamic development post meiosis did not reveal any apparent deviation from wt (Figure S3A). DAPI stained one-nucleated microspores were released from the tetrads (Figures S3A and S3B), but a variable number did not stain or hydrate in DAPI solution, indicating degeneration. After the first mitosis, some DAPI-stained microspores deviated slightly from wt in that generative nuclei appeared less compact and occasionally with two instead of one nucleolus (Figure S3C). At the trinucleate stage typically 25% of the pollen were DAPI stained (*n* = 65) compared to >99% in wt (n = 1290) (Figure S3D; viability was confirmed by Alexander staining.)

Especially in older flowers, dehiscence appeared to be delayed (Figure 4Bi). However, a low frequency of dehiscence occurred (Figure 4Bii and Figure S2A), releasing trinucleate pollen that could produce a pollen tube and appeared to be fully functional (Figure 4Biii).

### *pASHH2:GUS* is expressed in the tapetum layer and in post-meiotic pollen

*pASHH2:GUS* plants were used to correlate the defects in anther and pollen development to *ASHH2* expression patterns. No GUS expression was detected in the developing floral organ primordia or in pollen tetrads (Figure 4C and Figure 4D, insert). During early post-meiosis (floral stage 9) a weak signal could be detected in floral organs that persisted and increased in intensity at later stages (Figure 4D and 4E). Expression was found in anthers, as well as the gyneocium and mid veins of petals and sepals (Figure 4E). At stage 12, however, *pASHH2:GUS* was strongly expressed specifically in the maternal sporophytic tapetum layer accompanied by a less intense expression in the microspores (Figures 4F and 4G).

### Exine deposition is affected in *ashh2* pollen

As the tapetum layer contributes to pollen-wall synthesis and sporopollenin deposition, *ashh2* pollen grains were inspected for changes in the exine layer using scanning electron microscopy (SEM) (Figures 4H and 4I). Wt pollen grains have an oval shape with two parallel furrows (Figure 4Hi) and an outer ordered exine lattice mainly composed of polymerized sporopollenin (Figure 4Hii). All four *ashh2* alleles had abnormal pollen grains with overall irregular shape and an exine pattern deviating from the even reticulation of the wt (Figures 4I, Figure S4 and Table S3). The ridges (muri) were often flattened and more irregular with smaller spaces (lumina) that in some cases seem to be filled with material (Figures 4Iiii), and the reticulate tectum was in many cases incomplete or even totally absent giving the exine layer a spotted appearance (Figure 4Iiv and Figure S4).

### Tapetal development is distorted in *ashh2* mutant plants

**Figure 5:**
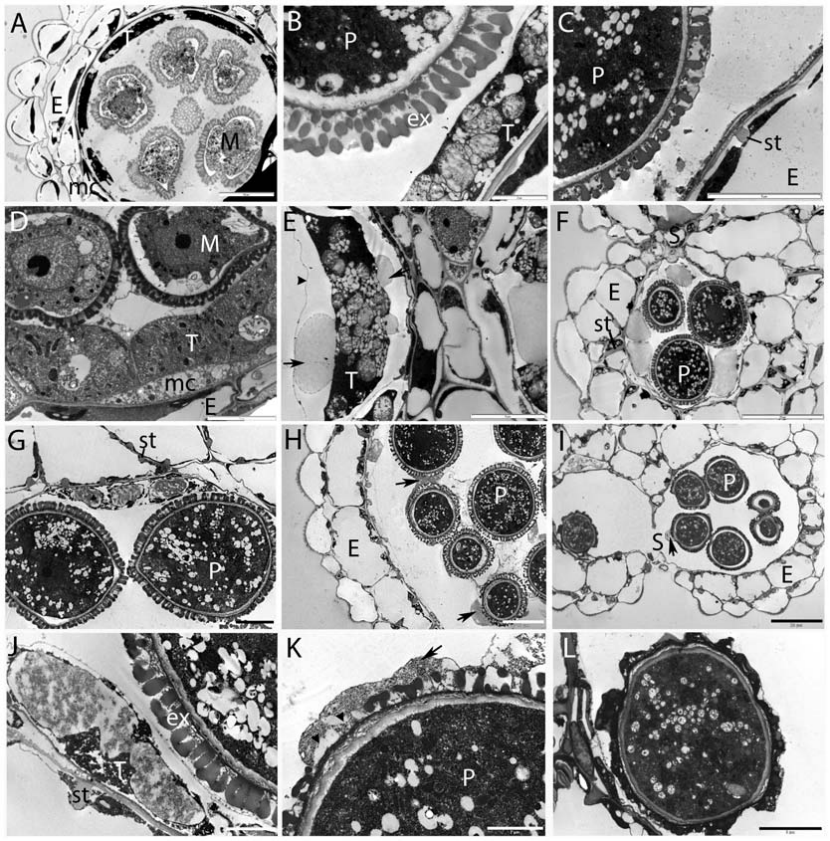
TEM analysis of tapetal and pollen development in *ashh2* and wt. (A) Early Ring Stage with free microspores (M) in wt. Note the endothecium (E), tapetum (T) and intact middle cell layer (mc). (B) Pollen Mitosis I to Pollen Mitosis II in wt, degeneration of the middle cell layer has occurred. Note exine (ex) with visible defined bacula. (C) Immediately prior to dehiscence in the wt. Tapetal degeneration and deposition of pollen coat has occurred and endothecium (E) secondary thickening is visible. (D) Early Ring Stage in the *ashh2-1* mutant displaying abnormal tapetal (T) enlargement. (E) Pollen Mitosis I to Pollen Mitosis II of *ashh2-1* disclosing abnormal accumulations of lipid-like material in membrane bound vesicles (arrows) within the tapetal cells (T). Note that normal tapetal degeneration fails to occur and that the tapetal membrane is clearly visible (arrowhead). (F) Prior to dehiscence in the *ashh2-1* mutant, normal endothecium secondary thickening (st) and thinning of the stomium (S) is seen. However the tapetal cells (T) are still present and release of the globular material previously visible (Figure 6E) has not occurred, suggesting that tapetal development is impaired and delayed. (G and J) Pollen Mitosis I to Pollen Mitosis II in the *ashh2-2* mutant, note secondary thickening (st) of the endothecium and that degeneration of the middle cell layer has occurred. Exine formation (ex) with well-defined bacula is visible, however, the tapetum (T) appears enlarged with an abnormal accumulation of wall materials. (H and K) As pollen mitosis progresses in *ashh2-2* abnormal deposition of pollen wall material is evident (arrow), resulting in agglutination of the immature pollen grains. In some cases a deficiency of the exine sculpturing is observed (arrowheads). (I and L) Prior to dehiscence in *ashh2-2* normal breakdown of the stomium occurs, however abnormal pollen wall development is observed (arrow), resulting in some cases in extreme malformation of the pollen wall (L). Bars in A, H: 10 µm; C, D, E, G, L: 5 µm; B, J, K: 2 µm; F, I: 20 µm.

Mutant anther development was studied in more detail using transmission electron microscopy (TEM), as many *ashh2* anthers showed locules with a number of aberrations (Figure S2). During Early Ring Stage in the wt (Figure 5A) the microspores are free and the tapetum is actively synthesizing wall materials. In locules of *ashh2-1* and *ashh2-2* anthers, abnormal tapetal enlargement could be found at the corresponding stage (Figures 5D, 5G and 5J). During Pollen Mitosis I (PMI) to PMII (Figure 5B) active secretion of wall material occurs by the wt tapetum to form the exine layer, and the tapetal membrane degenerates. In the corresponding stage in *ashh2-1* (Figure 5E), globular material accumulated in membrane-bound vesicles (arrow in 5E) that fused to form the major components of the tapetum (Figure 5F). In the *ashh2-2* allele abnormal accumulation and deposition of pollen wall material were evident (Figures 5G and 5J and arrow in Figures 5H and 5K) leading to agglutination of the immature pollen grains. Immediately prior to dehiscence in the wt, the tapetum has fully degraded and the pollen coat has been deposited (Figure 5C). In contrast, the mutant tapetum and also the tapetal membrane persisted longer (Figure 5F and arrowhead in Figure 5E). In some cases severe deficiencies in the exine sculpturing and abnormal pollen wall development were observed (Figures 5I and 5L), consistent with the results from the SEM analysis (Figures 4I and Figure S4). Contrary to the distorted tapetum development, secondary thickening of the endothecium, degeneration of the middle cell layer, as well as stomium breakdown appeared normal in the mutant (Figure 5).

### Mutation in *ASHH2* leads to substantial changes in inflorescence gene expression

**Figure 6:**
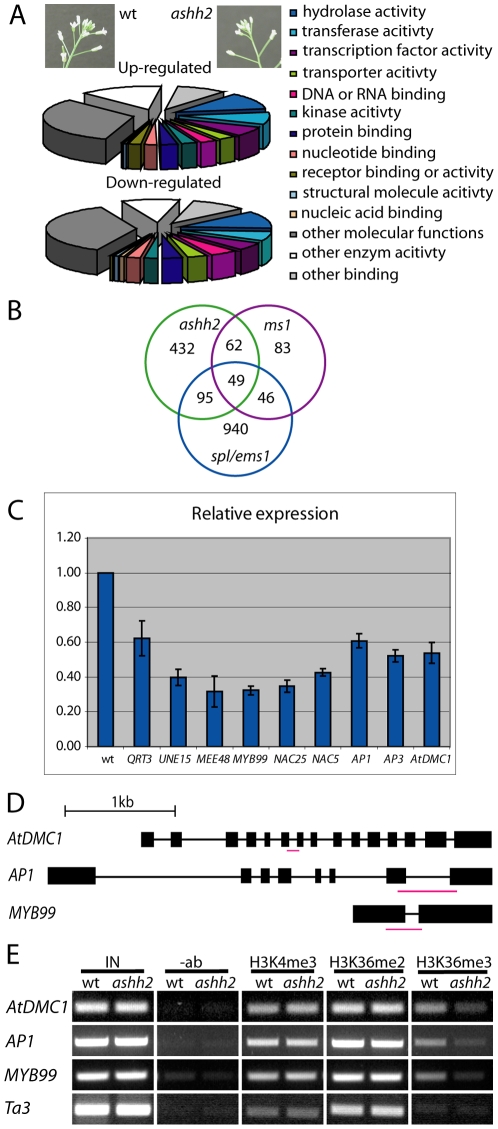
Changes in gene expression and histone tail methylation in the *ashh2-1* mutant. (A) Functional GO annotation of genes differentially expressed in *ashh2-1* inflorescences relative to wt. The inserts depict the developmental stage of the main inflorescences used in the microarray experiment. (B) Number of co-down-regulated genes in the *ashh2-1* mutant (>1.6-fold change), *ms1* mutant (>2-fold change) and *spl*/*ems-1* mutant (>1.6-fold change). (C) Confirmation of down-regulation of selected genes using real-time reverse transcriptase PCR. Wt levels were set to 1. (D) Gene structures of *AtDMC1*, *AP1* and *MYB99*. Boxes indicate exons, thin lines introns. The positions of the fragments tested in the ChIP analyses are shown as red lines. (E) Representative results of ChIP analyses on the selected down-regulated genes shown in (D) using antibodies against H3K4me3, H3K36me2 and H3K4me3. *Ta3* was used as a control. IN – input; –ab – without antibodies.

Total RNA from wt and *ashh2-1* young flowers and buds was used in a microarray experiment encompassing oligonucleotides representing ∼27 000 unique genes aimed at identifying target genes for ASHH2 (see Materials and Methods) (Figure 6A). When controlling the false discovery rate (FDR) [17] in the statistical analysis at a 0.01 level, and using an absolute log_2_-ratio >0.7 as cut-off (i.e. up- or down-regulation 1.6 times), 348 genes were up-regulated and 637 down-regulated in *ashh2-1* compared to wt.

These gene sets were analyzed with the TAIR GO annotation tool (http://www.arabidopsis.org/tools/bulk/go/index.jsp). A higher proportion of the upregulated genes were related to biotic or abiotic stimulus or stress processes (11%) than the down-regulated genes (5%) (Figure 6A and Table S2), and only one of the up-regulated genes, *MATERNAL EFFECT EMBRYO ARREST 38 (MEE 38)*, is known to be involved in reproductive processes [18]. In contrast, a number of the down-regulated genes are known to be involved in such processes (Table 3), e.g. *ATDMC1* needed for meiosis [19]; *ABORTED MICROSPORES (AMS)* and *MALE STERILE 2 (MS2)*, involved in tapetum function and anther dehiscence [20], [21].

**Table 3:** Down-regulated genes in *ashh2* inflorescences encoding transcription factors and factors involved in development.

AtGID	Name	Reference	Expression^a^	Log_2_-ratio	Fold down
At1g02800	CEL2	Yung et al., Plant J 17: 203–208 (1999)	shoot apex, carpels	−1.12	2.18
At1g08320	bZIP TF	Jakoby et al., Trends Plant Sci 7: 106–111 (2002)	flower	−0.99	1.99
At1g18960	MYB TF	-	n.a.	−1.50	2.83
At1g26310	CAL	Kempin et al., Science 267: 522–525 (1995)	flower	−0.53	1.45
At1g26780	MYB117	Stracke et al., Curr Opin Plant Biol 4: 447–456 (2001)	shoot apex	−0.76	1.70
At1g31760	BAF60b of Swib	-	shoot apex, carpels	−1.36	2.56
At1g35490	bZIP TF	-	pollen	−0.76	1.70
At1g61110	AtNAC025	Wellmer et al, Plant Cell, 16: 1314–1326 (2004)	tapetum	−1.46	2.75
At1g67710	ARR11	Mason et al., Plant Cell, 17: 3007–3018 (2005)	n.t.	−1.10	2.15
At1g68190	zinc finger	-	n.t.	−1.46	2.75
At1g69180	CRC	Bowman & Smyth, Develop 126: 2387–2396 (1999)	shoot apex, carpels	−0.77	1.70
At1g69120	AP1	Mandel et al., Nature 360: 273–277 (19942)	flower	−0.43	1.35
At1g69940	AtPPME1	Tian et al., Dev Biol 294: 83–91 (2006)	n.a.	−1.07	2.10
At1g71130	AtERF#070	Nakano et al., Plant Physiol 140: 411–432 (2006)	n.t.	−1.07	2.10
At1g77080	MAF1	Ratcliffe et al., Plant Physiol 126: 122–132 (2001)	n.t.	−2.44	5.43
**At1g77300**	**ASHH2**	Present work		−1.25	2.38
At2g01200	MEE10/IAA32	Pagnussat et al., Development 132: 603–614 (2004)	n.t.	−1.03	2.04
At2g01760	ARR14	Mason et al., Plant Cell, 17: 3007–3018 (2005)	n.t.	−0.83	1.78
At2g03740	LEA-domain	-	stamens	−1.42	2.68
At2g03850	LEA-domain	-	stamens	−1.43	2.69
At2g04630	RBP6	Devaux et al., Mol Biol Cell 18: 1293–1301 (2007)	n.t.	−1.24	2.37
At2g07690	CDC46 homologue	-	shoot apex, carpels	−0.93	1.91
At2g15400	RPB36B	Larkin & Guilfoyle, J Biol Chem 272 19: 12824–12830 (1997)	n.t.	−0.77	1.70
At2g16210	B3 TF	-	shoot apex, carpels	−1.23	2.35
At2g16910	AMS	Sorensen et al., Plant J 33: 413–423 (2003)	stamens	−1.46	2.75
At2g19770	Profilin PFN4	Christiensen et al., Plant Journal 10: 269–279 (1996)	pollen	−1.14	2.21
At2g24650	B3 TF	-	shoot apex, carpels	−0.87	1.82
At2g24700	B3 TF	-	n.a.	−0.82	1.77
At2g31220	bHLH TF	Heim et al., Mol Biol Evol 20: 735–747 (2003)	n.a.	−1.03	2.04
At2g33810	SPL3	Wu & Poethig, Develop 133: 3539–3547 (2006)	n.t.	−1.04	2.05
At2g35310	B3 TF	Wellmer et al., PLoS 2 (7): e117 (2006)	buds	−0.72	1.65
At2g42660	MYB TF	-	pollen	−1.11	2.15
At2g42940	AHL16	Fujimoto et al., Plant Mol Biol 56: 225–239 (2004)	flower	−1.23	2.35
At2g46020	BRM	Hurtado et al., Plant Mol Biol 62: 291–304 (2006)	n.t.	−0.99	1.98
At2g46770	NST1	Mitsuda et al., Plant Cell 17: 2993–3006 (2005)	stamens	−0.93	1.90
At2g47040	VGD1	Jiang et al., Plant Cell 17: 584–596 (2005)	pollen	−0.79	1.73
At3g06220	B3 TF	-	shoot apex, carpels	−1.18	2.26
At3g11980	MS2	Aarts et al., Plant J 12: 615–623 (1997)	stamens	−1.86	3.64
At3g12040	DNA glycosylase	-	n.t.	−0.82	1.77
At3g18960	B3 TF	-	seeds, shoot apex	−0.73	1.66
At3g22880	ATDMC1	Couteau et al., Plant Cell 11: 1623–1634 (1999)	shoot axep, carpels	−0.73	1.66
At3g24650	ABI3	Parcy et al., Plant Cell 6: 1567–1582 (1994)	seeds	−1.09	2.12
At3g42960	ATA1	Lebel-Hardenack et al., Plant J. 12: 515–526 (1997)	stamens	−1.32	2.50
At3g43920	DCL3	Xie et al., PLoS Biol 2(5): E104 (2004)	shoot apex, carpels	−0.90	1.87
At3g53310	B3 TF	Wellmer et al., PLoS 2 (7): E117 (2006)	shoot apex, carpels	−1.09	2.13
At3g54340	AP3	Jack et al., Cell 76: 703–716 (1994)	shoot apex, carpels	−0.89	1.85
At3g63440	AtCKX7	Werner et al, Plant Cell 15: 2532–2550 (2003)	shoot apex	−1.00	2.00
At4g00870	bHLH TF	Heim et al., Mol Biol Evol 20: 735–747 (2003)	shoot apex, carpels	−0.73	1.66
At4g01580	B3 TF	-	n.a.	−0.79	1.73
At4g07400	VFB3	Schwager et al., Plant Cell 19: 1163–1178 (2007)	n.t.	−0.88	1.84
At4g13230	LEA-domain	-	pollen	−1.35	2.55
At4g13560	UNE15	Pagnussat et al., Development 132: 603–614 (2004)	pollen	−1.29	2.45
At4g14080	MEE48	Pagnussat et al., Development 132: 603–614 (2004)	flower	−1.69	3.22
At4g17280	similar to AIR12		seeds	−0.88	1.84
At4g19230	CYP707A1	Okamoto et al., Plant Physiol 141: 97–107 (2006)	stamens	−0.77	1.71
At4g20050	QRT3	Rhee et al., Plant Physiol 133: 1170–1180 (2003)	stamens	−1.22	2.33
At4g23570	SGT1A	Azevedo et al.,EMBO J 25: 2007–2016 (2006)	n.t.	−0.88	1.84
At4g23660	AtPPT1	Okada et al., Plant Mol Biol 57: 567–577 (2004)	flower	−0.88	1.85
At4g29340	Profilin PFN3	Christiensen et al., Plant Journal 10: 269–279 (1996)	pollen	−1.25	2.38
At4g30860	ASHR3	Thorstensen et al., Plant Mol Biol 66: 47–59	stamens	−0.61	1.53
At4g31620	B3 TF	-	shoot apex	−0.74	1.67
At4g34400	B3 TF	-	shoot apex	−0.96	1.95
At4g34710	ADC2	Urano et al., FEBS Letters 579: 1557–1564 (2005)	n.t.	−0.80	1.75
At5g13930	CHS/TT4	Brown et al., Plant Physiol 126: 524–535 (2001)	n.t.	−0.73	1.66
At5g16970	At-AER	Mano et al., Plant Physiol 139:1773–1783 (2005)	n.t.	−0.91	1.87
At5g20240	PI	Goto and Meyerowitz, Genes Dev 8: 1548–1560 (1994)	flower	−0.53	1.44
At5g23405	HMG1/2 PROTEIN	Grasser et al., J Mol Biol 358: 654–64 (2006)	shoot apex, flower	−0.71	1.64
At5g28390	RNA-recogn. motif	-	n.a.	−0.91	1.88
At5g37890	SINAT5	Kim et al. Mol Cells 21: 389–394 (2006)	shoot apex, flower	−0.77	1.70
At5g40260	RPG1	Guan et al. Plant Physiol 147: 852–863 (2008)	flower	−1.46	2.74
At5g54340	zinc finger	-	n.a.	−0.73	1.66
At5g61430	AtNAC5	Yang et al. Plant Cell 19: 3530–3548 (2007)	anther	−058	1.49
At5g62320	MYB99	Wijeratne et al. Plant J 52: 14–29 (2007)	anther	−1.22	2.33
At5g65050	MAF2	Ratcliffe et al., Plant Cell 15: 1159–1169 (2003)	n.t.	−0.87	1.83
At5g65060	MAF3	Ratcliffe et al., Plant Cell 15: 1159–1169 (2003)	n.a.	−1.27	2.42
At5g65070	MAF4	Ratcliffe et al., Plant Cell 15: 1159–1169 (2003)	seeds	−0.49	1.40
At5g65080	MAF5	Ratcliffe et al., Plant Cell 15: 1159–1169 (2003)	seeds	−1.75	3.37

a Expression patterns have roughly been grouped by using AtGenexpress (AtGE Development). n.a. – gene not on Affimetrix micro arrays. n.t. – expression shows little tissues-specificity.

Twenty-three and 35 genes, respectively, encoding putative transcription factors (TFs) were up- and down-regulated (Figure 6A), however, several of these have undefined functions (Table 3). The most strongly down-regulated gene was the *FLC*-related *MADS-BOX AFFECTING FLOWERING 1* (*MAF1*), which has been reported down-regulated in *ashh2* seedlings [6], [7], [14], [22]. Likewise the highly similar and clustered genes *MAF2-MAF5* [14], [22] were all down-regulated in the inflorescence sample (Table 3).

### A number of genes are co-down-regulated in *ashh2* and *ms1* and/or *spl/ems1* mutants

A network of genes controls anther development and function, including *SPOROCYTELESS (SPL)* which act very early in anther development, *EXCESS MICROSPOROCYTES 1* (*EMS1*) involved in early tapetal initiation, and *MALE STERILE 1* (*MS1*) needed for tapetal development and function. As the *ashh2* mutants have a clear defect in pollen production we compared *ashh2* mutant microarray data with array data for *spl*, *ems1* and *ms1* mutants [23], [24]. The expression levels of these genes themselves were not significantly altered in the *ashh2* background, indicating that *ASHH2* is not acting upstream of these genes, or acts in independent pathways. Only one gene was up-regulated in both the *ashh2* and *ms1* microarrays and only ten genes were co-upregulated in the *ashh2* and *spl/ems1* microarrays. In contrast, a significant number of genes showed coordinate down-regulation, suggesting downstream involvement in common pathways. Out of 1030 genes down-regulated in both *spl* and *ems1*, 144 (log_2_>0.7) were also down-regulated in *ashh2* (Figure 6B). Of the 240 genes down-regulated more than two fold in *ms1* [24], 46.3% (111 genes) were also down-regulated in *ashh2* mutant inflorescences (Table S4). These encompass tapetum-specific genes encoding oleosins and lipases that together make up 90% of the detectable pollen coat protein [25], as well as the genes encoding the transcription factors MYB99, AtNAC25 and AtNAC5 suggested to be direct targets of MS1 [24], [26].

### Genes associated with the *ashh2* phenotype show a reduction in gene expression and H3K36 trimethylation

Genes that were down-regulated in the microarray experiment and potentially involved in different aspects of the *ashh2* mutant phenotype showed consistent expression changes by real-time PCR (Figure 6C, Table 3). The expression levels of the *AP1* and *AP3* genes, encoding A and B floral organ identity factors, respectively, and the *QRT3* and *AtDMC1* genes, were reduced by 40 to 50%. *MEE48* and *UNE15* involved in female gametophyte development and function, as well as the tapetal TFs *MYB99*, *AtNAC5* and *AtNAC25* were down-regulated 60 to 70%.

Based on the ASHH2 protein's similarity to other ASH1-like proteins working as transcriptional co-activators [27] (Figure S5) and ChIP data from *ashh2* non-reproductive tissue [6], [7], [12], [13], we expected genes that are potential direct target of ASHH2 to be down-regulated and to have changes in the level of histone H3 methylation at K4 and/or K36 in *ashh2*. Chromatin was isolated from inflorescences of young wt and *ashh2-1* mutant plants, and precipitated using antibodies against H3K4me3, H3K36me2 and H3K36me3. Two to three biological replicas were used for each precipitation, and genes representing different aspects of the mutant phenotype (*AP1* – floral organ identify; *AtDMC1* – embryo sac development; *MYB99* – pollen development, Figure 6D) were selected for ChIP analysis. In all cases no changes were seen for H3K4me3 or H3K36me2 (Figure 6E and Figure S6). In contrast, substantial reduction of H3K36me3 was revealed (Figure 6E and Figure S6) both relative to the input and the Ta3 retrotransposon used as a control [7], [28]. This suggests that ASHH2 regulates expression of these genes via H3K36 trimethylation.

## Discussion

Mutations in *ASHH2* lead to early flowering, have an obvious effect on shoot branching and were recently also shown to affect carotenoid composition (Figure 1H, Figure S1A and [6]–[8], [12], [13]. Here we have focused on the involvement of *ASHH2* in the regulation of organ identity in the flower, and its role in ovule and anther development.

### Floral homeotic changes correlate to down-regulation of homeotic genes in the first and second whorl

Floral organ identity is governed by the interplay between transcription factors expressed in the four whorls of the flower [29]. Mutations in whorl 1 and 2 expressed *AP1* or *AP2* genes leads to a spread of *AG* expression from whorl 3 and 4 and results in carpels in whorl 1 and stamen in whorl 2. Additional mutations in whorl 2 and 3 expressed *AP3* or *PI* genes show a further conversion to carpeloid organs [30]. The observed homeotic changes of the floral organs in the *ashh2* Col alleles as well as the *esf1-1* L*er* allele, with carpeloid organs in whorls 2 and 3, or conversion of sepals to carpels and petals to stamen (Figures 1K to 1M, Table S1), are in accordance with down-regulation of *AP1*, *AP3* and *PI* in *ashh2* inflorescences (Table 3, Figure 6C).

In mutants defective in the *ATX1* gene encoding a H3K4 HMTase, floral homeotic genes are also down-regulated [10]. This may be an indirect effect, as H3K4me3 marks on the *AP1* gene did not decrease in the *atx1* flowers [31], [32]. In contrast, the down-regulation of *AP1* in the *ashh2* mutant was correlated with a reduction of the H3K36me3 level (Figure 6C and E).

### *ASHH2* is required for normal embryo-sac development

Reciprocal crosses of *ASHH2/ashh2* heterozygous plants indicated that *ASHH2* is needed for normal development or function of the haploid gametophytes (Figures 2A and 2B, Table 2). The sporophytic effect of a mutation in *ASHH2* is, however, much more pronounced (Figures 2A and 2B), and has been the main focus in this work.

In homozygous *ashh2* plants, mature ovules were devoid of an embryo sac (Figure 3B) and the majority of the embryo sacs arrested before the first syncytial nuclear division (Figure 3C). At premature stages, however, embryo sacs undergoing nuclear division could be observed (Figures 3D to 3I), and in these cases the embryo sacs were often protruding out of the ovule between underdeveloped integuments (Figures 3G to 3I). This could suggests that when nuclear proliferation takes place, the delayed elongation of outer integuments force the protruding embryo-sac structure out, causing it to eventually degenerate or “burst”. Such expulsion of the female gametophyte has been previously described for the *clotho* (*clo*) mutant, and is suggested as a mechanism to hamper allocation of maternal resources to sterile tissue [33]. However, these researchers demonstrated expulsion of mature female gametophytes 3 days after emasculation, and our results indicate that this mechanism also can act at earlier developmental stages. *pASHH2:GUS* expression was found both in the integuments and in the young nucellus where the MMC form (Figures 2E and 2F), supporting a function for ASHH2 both in ovule development and pre- or post-meiotic sporophytic contribution to the generation of the female gametophyte. Occasionally, however, normal progression through female meiosis occurred in the mutant since homozygous lines could generate a low frequency of vital seeds.

There are hundreds of mutants defective in embryo sac formation [34], but only in a few cases the affected genes have been identified. The observed *ashh2* phenotype resembles that of the mutants *pretty few seeds 2* (*pfs2*), *sporocyteless/nozzle* (*spl/nzz*) and *atdmc1* [19], [35], [36]. Only *atdmc1* was down-regulated in our microarray experiment (Table 3 and Figure 6C). We consider it likely, however, that additional down-regulated, yet unidentified genes also contribute to the *ashh2* mutant embryo sac and ovule phenotype.

The *atdmc1* mutant is defective in female and male sporogenesis due to severely distorted chromosomal segregation, but still allows a low seed set (1.5% of wt) [19], [37]. In *ashh2,* down-regulation of *atdmc1* possibly takes place only in the female reproductive organ as *pASHH2:GUS* expression was not detected in pollen tetrads (insert Figure 4D), and meiosis appeared normal (Figure S3). Down-regulation of *AtDMC1* in the *ashh2* female organ is, however, likely since *pASHH2:GUS* was detected where the MMC form (Figure 2E). The down-regulation is accompanied with a reduction of the H3K36m3 level (Figure 6C and E).

At the fully developed ovule stage the *spl/nzz* mutant phenotype resembles that of *ashh2*, but in contrast to *ashh2, spl/nzz* is characterized by the absence of a MMC at an early stage of ovule development, thus preventing embryo sac formation [36].

### *ASHH2* is required for normal anther differentiation, tapetum development and pollen maturation

On the male side mutation of *SPL/NZZ* result in a lack of initial division of the archesporial cells that leads to the primary parietal layer, thus pollen sacs and PMCs do not differentiate [36]. This resembles the *ashh2* phenotype, where about a quarter of the anthers do not develop proper locules (Figure 4B and Figure S2). However, we have no indications that *ASHH2* works directly through *SPL*/*NZZ* as no down-regulation of this gene was seen in our microarray experiment, and locule development and meiosis occur, albeit always producing a reduced number of microspores (Figures 4A and 4B). The defect in pollen production is likely to result from aberrations in tapetal layers from PMI onwards with accumulation of vesicles, abnormalities in the secretion of wall materials and delayed degeneration (Figures 5D to 5L and Figure S2). By the dehiscence stage severe distortions and collapse of pollen wall could be observed with deformed or absent bacula and tectum (Figures 5L, 4I and Figure S4). This may either be due to delayed cell wall deposition or altered wall composition as the expression data showed down-regulation of pollen coat-related genes, e.g. the oleosin genes (Table S4). A similar down-regulation of these genes is seen in the *ms1* mutant, which also presents defective late pollen wall and coat development [38]. Delay in tapetal development was not necessarily reflected in delay of other aspects of anther development, like dehiscence (Figure 5, Figure S2A), suggesting a lack of synchrony and coordinated regulation in the development of the different anther tissues.

### Transcriptional profiling suggests that *ASHH2* has a broad effect on transcription factors controlling development

Ten percent of the genes down-regulated in *ashh2* inflorescences encoded either transcription factors or DNA or RNA binding proteins (Figure 6A). Many of these, e.g. the B3 TFs, have not yet been assigned a specific biological function (Table 3), and the significance of their down-regulation in the *ashh2* mutant cannot be evaluated at present. Similarly, the function of the most likely redundant *MAF* genes in the inflorescence is unknown.

In contrast to genes upregulated in *ashh2* inflorescences, there is a substantial overlap between genes down-regulated in the *ms1* and the *ashh2* mutants. This overlap might reflect the sum of anther defects, however, all *ms1* affected genes are not down-regulated in the *ashh2* inflorescences, suggesting that the co-regulated subset is part of a genetic network controlled by both *ASHH2* and *MS1*. Down-regulated genes that are common for *ashh2* and *spl/nzz*, but not *ms1*, e.g. *MS2*, *AMS* and its putative interacting partner ASHR3 [20], [21], [39], can be assumed to partake in genetic networks operating earlier than, or in parallel to, *MS1* during anther development.

Since the microarray suggested that *MS1* and *SPL/NZZ* themselves are not down-regulated in the *ashh2* mutant one hypothesis could be that the ASHH2 protein works together with MS1 and/or SPL/NZZ to control their direct target genes. Consistent with this hypothesis is the down-regulation in *ashh2* background of the presumed direct targets of MS1, the transcription factors MYB99 and AtNAC25 [24], [26] (Figure 6C), as well as reduction of H3K36me3 on MYB99 chromatin (Figure 6E).

### Changes in histone tail marks in *ashh2* mutant plants suggest ASHH2 to work via H3K36me3 in reproductive development

So far the exact substrate and product specificities of ASHH2 have not been demonstrated *in vitro*, possibly because some other unknown modification on the histone tails is a prerequisite for ASHH2's activity. ASHH2 has been implicated in H3K4me3 methylation on the *CAROTENOID ISOMERASE* gene and in the *FLC* promoter region [6], [13]. The latter has been suggested to be due to collaboration between ASHH2 and ATX1/ATX2 [40]. ASHH2 seem to be of more general importance for H3K36 methylation as substantial reduction in the global levels of H3K36me2 and me3, as well as H3K36me3 reduction in all gene specific regions tested so far in *ashh2* mutants have been shown [7], [12], [14]. The reduced H3K36me3 levels we have found for *AtDMC1*, *AP1* and *MYB99* in *ashh2* mutant inflorescences (Figure 6E) are consistent with a role for ASHH2 in H3K36 trimethylation.

The amino acid sequence of the SET domain and surroundi ng regions of ASHH2 have high similarity to H3K36me3 methyltransferases in yeast (Set2), *Drosophila* (dSet2) and mammals (Setd2/HYBP) [41]–[44] (Figure S5), which have been implicated in inhibition of transcriptional initiation within coding regions, DNA replication check-pointing, prevention of spreading of neighboring heterochromatin, and involvement with pre-mRNA splicing [45], [46]. These Set2 homologues interact with the C-terminal domain (CTD) of DNA Polymerase II via their Set2 Rpb1-interacting (SRI) region [47], which is not conserved in plant ASHH2 homologues. Thus it remains to be seen whether ASHH2 functions in the same way as any of these enzymes in its control of reproductive organ formation and other aspects of *Arabidopsis* growth and development.

## Materials and Methods

### Plant material

*Arabidopsis* plants were normally cultivated in growth chambers at 20°C for 8h of dark and 16 h of light (100 µE·m^−2^ s^−1^). The T-DNA insertion lines SALK_65480, SALK_026442, SALK_36941 and SALK_040477 were genotyped to establish homozygous lines, and these were designated *ashh2-1*, *ashh2-2, ashh2-5* and *ashh2-6*. *ashh2-1* and *-2* are identical to lines designated *sdg8-1* and -*2* by [7]. The exact positions of the T-DNA insertions were identified from sequenced PCR products of the T-DNA/plant DNA junctions.

### *pASHH2:GUS* lines

An *ASHH2* promoter fragment encompassing 1211 bp upstream of the ATG start codon was amplified using the primers prom-GUS Ashh2 attB1 and prom-GUS Ashh2 attB2 and introduced into the pDONR/Zeo Gateway entry vector and thereafter recombined into the promoterless GUS destination vector pMDC162 [48], generating the construct *pASHH2:GUS*. Transgenic *Arabidopsis* plants were produced by the floral dip method [49].

*GUS* staining and whole-mount clearing preparations of plant organs were performed as described [50].

### *n situ* hybridization

For *in situ* hybridization, an *ASHH2* 1 kb PCR product generated with the primers 2135L and 3115R was cloned into the vector pCR IV-TOPO (Invitrogen, Carlsbad, CA) and used as templates for dioxigenin-11-UTP labelling (Roche Molecular Biochemicals, Indianapolis, IN) of RNA probes. The *ASHH2* antisense RNA probe was synthesized using T3 RNA polymerase after linearization with *Not*I, and the sense control probe was synthesized with T7 RNA polymerase after linearization with *Spe*I. Plant fixation and *in situ* hybridization were performed according to [51].

### Microscopy

For Scanning Electron Microscopy (SEM) pollen grains from homozygous *ashh2* plants and wt pollen were harvested directly on a stub, coated with gold palladium in a sputter-coater, and inspected using a high-vacuum Scanning Electron Microscope (SEM) JEOL JSM 6400.

For Transmission Electron Microscopy (TEM) whole inflorescences were fixed and processed according to [38]. Individual buds were embedded in LR White resin (TAAB) and ultra-thin (50–70 nm) sections were cut and collected on uncoated 200 hexagonal mesh copper grids (TAAB). Sections were double stained with saturated uranyl acetate in 50% (v/v) ethanol and Reynolds lead citrate and examined under a FEI Tecnai 12 Biotwin TEM. Semi-thin sections (0.5 µm) were cut and stained with toluidine blue (0.05% w/v) for light microscopy observation of anthers using a Nikon OPTIPHOT.

For ovule and embryo sac morphology, material was fixed and processed as described previously [50] and analyzed by differential interference contrast light microscopy (DIC).

For anther and pollen morphology, whole inflorescences were fixed in 4% paraformaldehyde in 1x PBS, pH 6.9 at room temperature and 10 mmHg for 1 h, washed twice for 10 min in 1 x PBS. Single anthers were mounted in staining buffer (1xPBS, 5% DMSO, and 0.01% Tween 20) containing combinations of 0.1 mg/mL propidium iodide, 2 mg/mL DAPI, or 0.01% (w/v) aniline blue and inspected in a Zeiss Axioplan2 imaging microscope equipped with epifluorescence. Pollen viability was confirmed by Alexander staining [52].

### Microarray experiment and data analysis

Five biological replicas with 12 plants each, of both wt and the *ashh2-1* mutant were sown out on soil and grown under the following conditions; 22°C day and 18°C night, 16 h day length with 30 min adjustment of light to on and off, and 85 µE m^−2^ s^−1^ in light intensity. The inflorescences of each biological repeat were harvested in bulk at same developmental stage (Figure 6A) and total RNA was extracted using RNeasy midi kit (Qiagen).

Gene expression profiles for *ashh2* inflorescences were compared to their wt counterparts using two-color microarrays and statistical analysis according to [53]. Differentially expressed genes (Table S5) identified using the limma software package [54]. All parts of the data analysis were performed using R [55]. All data are MIAME compliant and that the raw data has been deposited in the MIAME compliant database GEO (http://www.ncbi.nlm.nih.gov/geo/query/acc.cgi?acc=GSE18325).

### Real-time quantitative PCR

Total RNA was treated with DNase I (Takara) followed by RNA Cleanup with RNeasy (Qiagen). Ten µg DNase treated total RNA were used for a double cDNA synthesis reaction with oligo-dT primers and SuperScript™ III Reverse Transcriptase (Invitrogen). RNA was removed from the cDNA by adding 2u of *E. coli* RNaseH (Takara). PCR reactions (5 µL diluted cDNA, 0.3 µM forward and reverse primers, 10.0 µL SYBR® Premix Ex Taq™ (Takara)) were performed on a LC480 Light Cycler (Roche). The cycling conditions were: 10 s 95°C followed by 45 cycles of 4 s 95°C, 20 s 60°C, 15 s 72°C. Experiments were performed in three biological replicates, two technical replicates and cDNA dilution series of 50 ng, 20 ng and 5 ng. Crossing point (CP) values were generated using 2nd derivative calculation software (LC480). Expression levels of target genes in the *ashh2* mutant were calculated relative to wt levels with normalization to *ACTIN2*, using the formula RE = E_target_ΔCPtarget (wt- *ashh2*)]/E_actin_[(ΔCPactin (wt – *ashh2*)], where target represents the target gene analyzed.

Gene specific primers for real-time PCR were designed using Roche Probe Library Primer Design or the Primer Design tool in the Vector NTI software (Invitrogen) (see Table S6).

### ChIP

ChIP was essentially performed as described in [56]. 1.5 g of inflorescences from young plants was put under vacuum for 40 min in 37 ml of 1% formaldehyde to crosslink the chromatin. After addition of 2.5 ml 2M Glycine, the inflorescences were put under vacuum for additional 5 min to stop crosslinking and then rinsed twice in water, frozen in liquid N2 and grinded in a mortar. After extraction from the inflorescences, the chromatin was sonicated for 4 min on setting H (15 sec on, 15 sec off) in a Bioruptor UCD-200 sonicator (Diagenode). Chromatin was precleared using Protein A agarose beads (Upstate) and immunoprecipitated over night using 5 µl of antibody against H3K4me3 (#07-473, Upstate), H3K36me2 (#07-369, Upstate) or H3K36me3 (ab9050, Abcam). Immunoprecipitated DNA was analyzed by RT-PCR using primers against *MYB99*, *AtDMC1* and *AP1* (Table S5). The *Ta3* retrotransposon was used as a control [7], [28] for equal precipitation of non-target chromatin with the different antibodies. Two to three biological replicas were used for each antibody. Two to three PCR replicas were used per primer set.

### Accession Numbers

*ASHH2* - At1g77300, *SPL/NZZ* – At4g27330, *EMS1* - At5g07280, *MS1* - At5g22260, *FLC* - At5g10140. Accession numbers of other genes discussed, are found in Table 3.

## Supporting Information

Figure S1 Phenotype of *ashh2* mutant plants.
(0.12 MB PDF)

Figure S2 Light microscopy of *ashh2-1* mutant anthers immediately prior to dehiscence.
(0.14 MB PDF)

Figure S3 Pollen meiosis in *ashh2* mutants.
(0.07 MB PDF)

Figure S4 Scanning electron micrographs of *ashh2-2*, *ashh2-5* and *ashh2-6* pollen grains and exine layers.
(0.69 MB PDF)

Figure S5 Alignment of the ASHH2 SET domain to SET domains of related proteins.
(0.41 MB PDF)

Figure S6 Changes in histone tail methylation in the *ashh2-1* mutant.
(0.09 MB PDF)

Table S1 Frequency of homeotic transformations in different *ashh2* alleles.
(0.03 MB PDF)

Table S2 Up-regulated genes in *ashh2* inflorescences encoding transcription factors and factors involved in development.
(0.10 MB PDF)

Table S3 Percentage of pollen showing abnormal exine layers.
(0.02 MB PDF)

Table S4 Genes down-regulated in *ashh2* inflorescences and *ms1* young buds.
(0.13 MB PDF)

Table S5 Up- and down-regulated genes in inflorescences from the *ashh2* mutant compared to wt.
(0.87 MB XLS)

Table S6 List of primers.
(0.13 MB PDF)
