# Supplementary material for: The ASH1 HOMOLOG 2 (ASHH2) Histone H3 Methyltransferase Is Required for Ovule and Anther Development in Arabidopsis
Source: PLoS One. 2009 Nov 12;4(11):e7817. doi: 10.1371/journal.pone.0007817 (PMC2772814; doi:10.1371/journal.pone.0007817)
Supplement: Figure S1 — Phenotype of ashh2 mutant plants. (0.12 MB PDF) [file pone.0007817.s001.pdf]

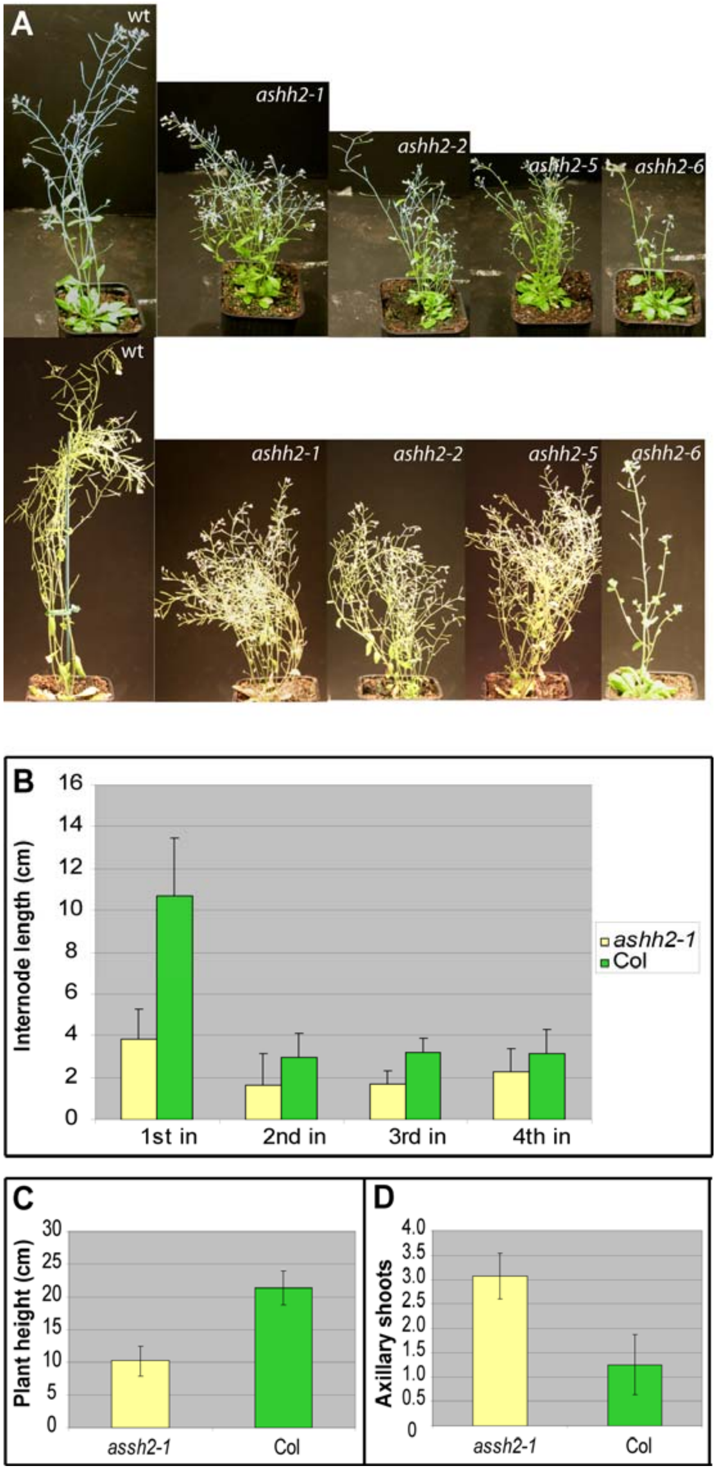


**Figure S1.**

**Phenotype of *ashh2* mutant plants.** (A) Wt plants (left) compared to the *ashh2* alleles at 40 DAG (upper panel) and 50 DAG (lower panel). Note that allele *ashh2-6* which has a T-DNA insertion in the promoter region, has a phenotype that differs both from wt and the other *ashh2* alleles that have T-DNA insertions in the transcribed region. (B) Length of the four first internodes in wt and *ashh2-1* plants. (C) Height of wt and *ashh2-1* plants. (D) Number of axillary shoots at 30 DAG for wt and *ashh2-1* plants.

Error bars indicate standard deviation. $n_{wt}$ = 12; $n_{ashh2\text{-}1}$ = 14.
