# Supplementary material for: The ASH1 HOMOLOG 2 (ASHH2) Histone H3 Methyltransferase Is Required for Ovule and Anther Development in Arabidopsis
Source: PLoS One. 2009 Nov 12;4(11):e7817. doi: 10.1371/journal.pone.0007817 (PMC2772814; doi:10.1371/journal.pone.0007817)
Supplement: Figure S2 — Light microscopy of ashh2-1 mutant anthers immediately prior to dehiscence. (0.14 MB PDF) [file pone.0007817.s002.pdf]

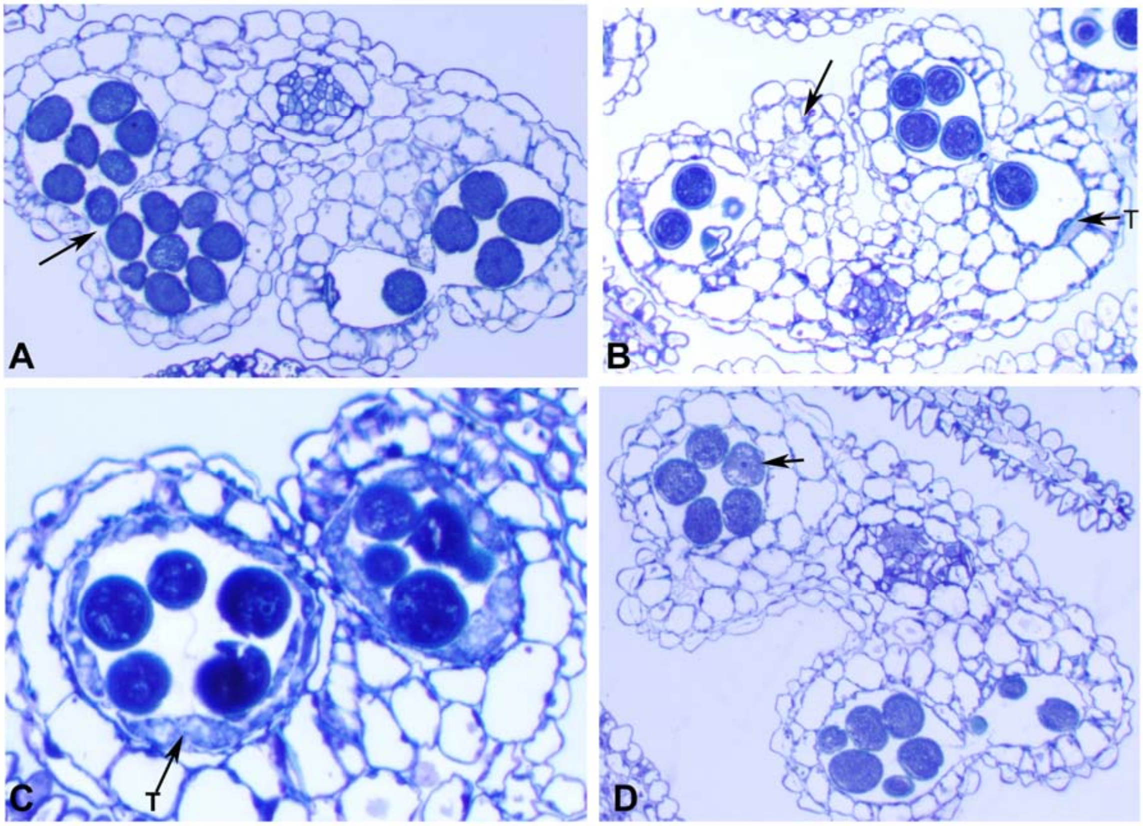

Figure S2. Light microscopy of *ashh2-1* mutant anthers immediately prior to dehiscence. (A) *ashh2-1* locule developing normally prior to anther opening, at the stage when the tapetal layer has been degraded in wt anthers. In some anthers of the *ashh2-1* mutant the stomium splits (arrow) followed by pollen dehiscence. (B), (C) and (D) *ashh2-1* locules with abnormal development, e.g. without any discernable organization and structure (arrow in B), with remains of the tapetum (T) (arrows in B and C), asynchronous development with some anthers showing advanced development, however with pollen with large levels of vesicles as seen in earlier stages (arrow in D).
