# Supplementary material for: The ASH1 HOMOLOG 2 (ASHH2) Histone H3 Methyltransferase Is Required for Ovule and Anther Development in Arabidopsis
Source: PLoS One. 2009 Nov 12;4(11):e7817. doi: 10.1371/journal.pone.0007817 (PMC2772814; doi:10.1371/journal.pone.0007817)
Supplement: Figure S3 — Pollen meiosis in ashh2 mutants. (0.07 MB PDF) [file pone.0007817.s003.pdf]

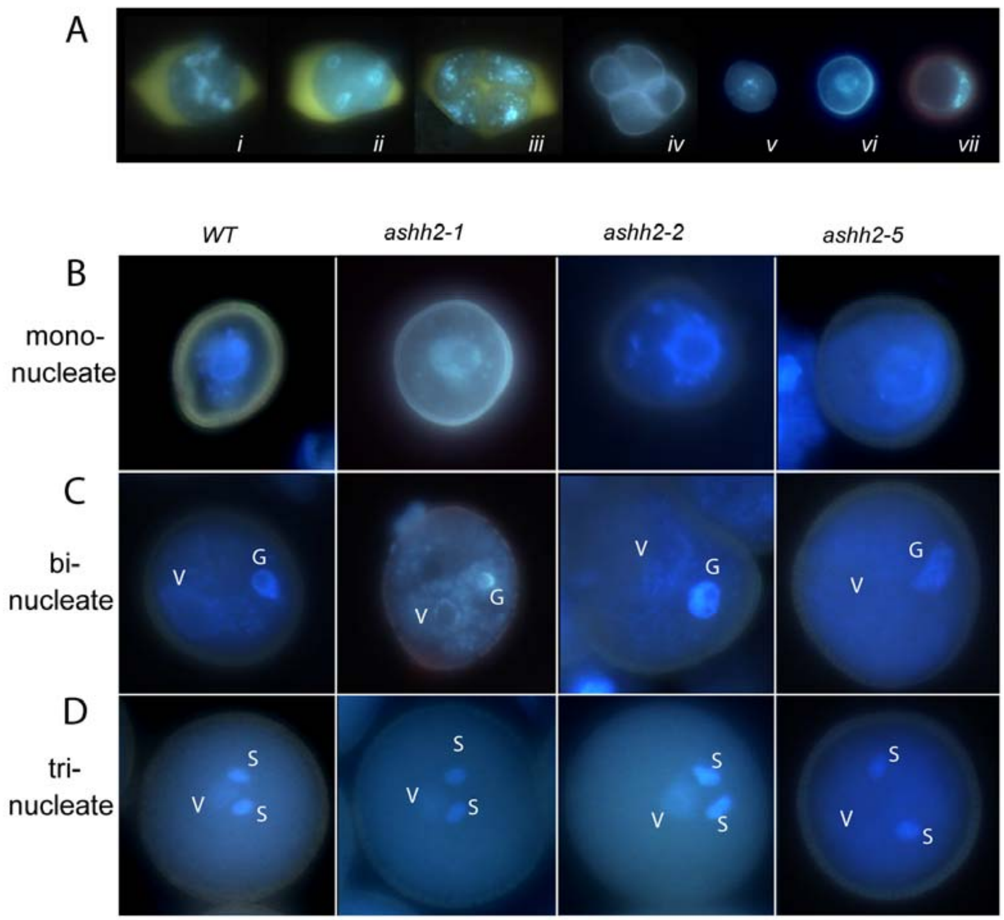

Figure S3. Pollen development in *ashh2*. (A) Fluorescent micrographs of meiosis and early progamic stages of pollen development in *ashh2-1* homozygous plants*.* Whole-mount staining with DAPI and Aniline Blue visualize nuclei (blue) and callose walls (yellow), respectively. (i) Meiosis at AnaphaseII, (ii) tetrad just before TelophaseII, (iii) tetrad after TelophaseII with meiotic products separated by callose walls , (iv) breakdown of the callose wall, (v) released mononucleate microspore, (vi) developing mononucleate microspore, (vii) ring-vacuolated mononucleate microspore. (B-D) Main stages of pollen development in wt and *ashh2-1*, *ashh2-2* and *ashh2-5*. Fluorescent micrographs of whole-mount staining with DAPI. (B) Mononucleate microspores before nuclear migration. (C) Bi-nucleate microspores after the first pollen mitosis (PMI) Vegetative cell (V), generative cell (G). Note, two nucleoli in *ashh2-2* generative nucleus.

(D) Tri-nucleate pollen after the second pollen mitosis (PMII). Sperm cells (S), vegetative cell (V). All pollen that showed DAPI staining appeared to be normal according to the corresponding wt stage. No developmental or cell cycle arrests could be observed.
