# Supplementary material for: The ASH1 HOMOLOG 2 (ASHH2) Histone H3 Methyltransferase Is Required for Ovule and Anther Development in Arabidopsis
Source: PLoS One. 2009 Nov 12;4(11):e7817. doi: 10.1371/journal.pone.0007817 (PMC2772814; doi:10.1371/journal.pone.0007817)
Supplement: Figure S4 — Scanning electron micrographs of ashh2-2, ashh2-5 and ashh2-6 pollen grains and exine layers. (0.69 MB PDF) [file pone.0007817.s004.pdf]

Figure S4. Scanning electron micrographs of *ashh2-2*, *ashh2-5* and *ashh2-6* pollen grains and exine layers.

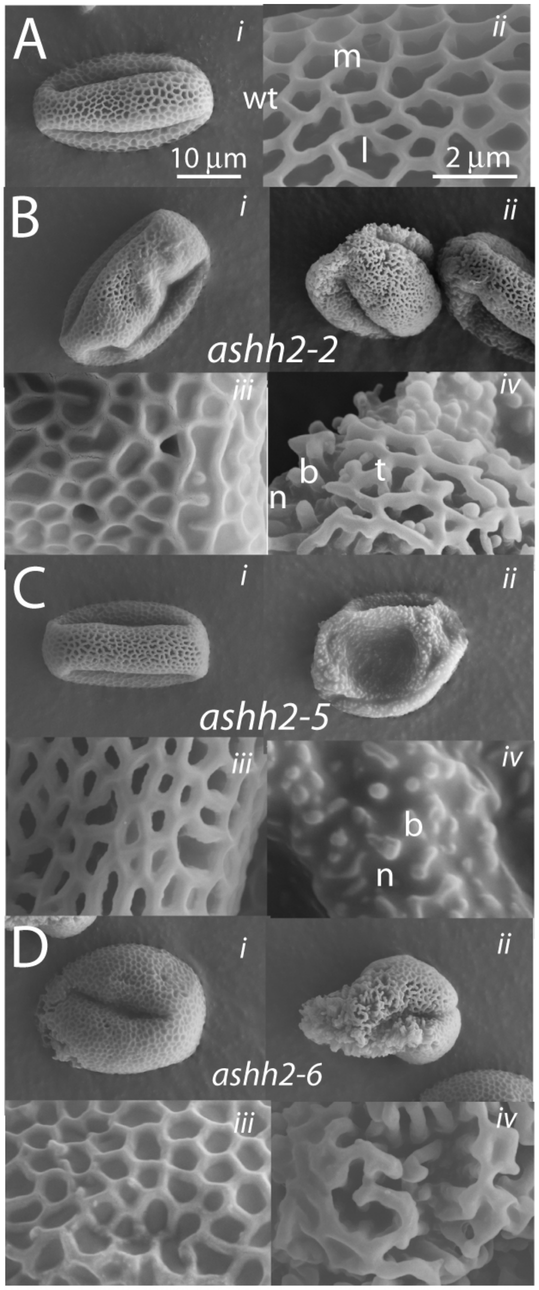

(A) Wt pollen (*i*) with sexine layer (*ii*) with regular ridges (muri – m) and spaces (lumina – l).

(B) *ashh2-2* pollen grains (*i, ii*) and exine layers with filled muris (*iii*), and in severe cases (*iv*) irregular and distorted textum layer (t).

(C) *ashh2-5* pollen grains (*i, ii*) and exine layers with thicker muris (*iii*), and in severe cases (*iv*) abscent tectum layer leading to disconnected bacula (b) and visible nexine layer (n).

(D) *ashh2-6* pollen grains (*i, ii*) and exine layers with filled muris (*iii*), and in severe cases (*iv*) irregular and distorted textum layer (t).
