# Supplementary material for: The ASH1 HOMOLOG 2 (ASHH2) Histone H3 Methyltransferase Is Required for Ovule and Anther Development in Arabidopsis
Source: PLoS One. 2009 Nov 12;4(11):e7817. doi: 10.1371/journal.pone.0007817 (PMC2772814; doi:10.1371/journal.pone.0007817)
Supplement: Figure S5 — Alignment of the ASHH2 SET domain to SET domains of related proteins. (0.41 MB PDF) [file pone.0007817.s005.pdf]

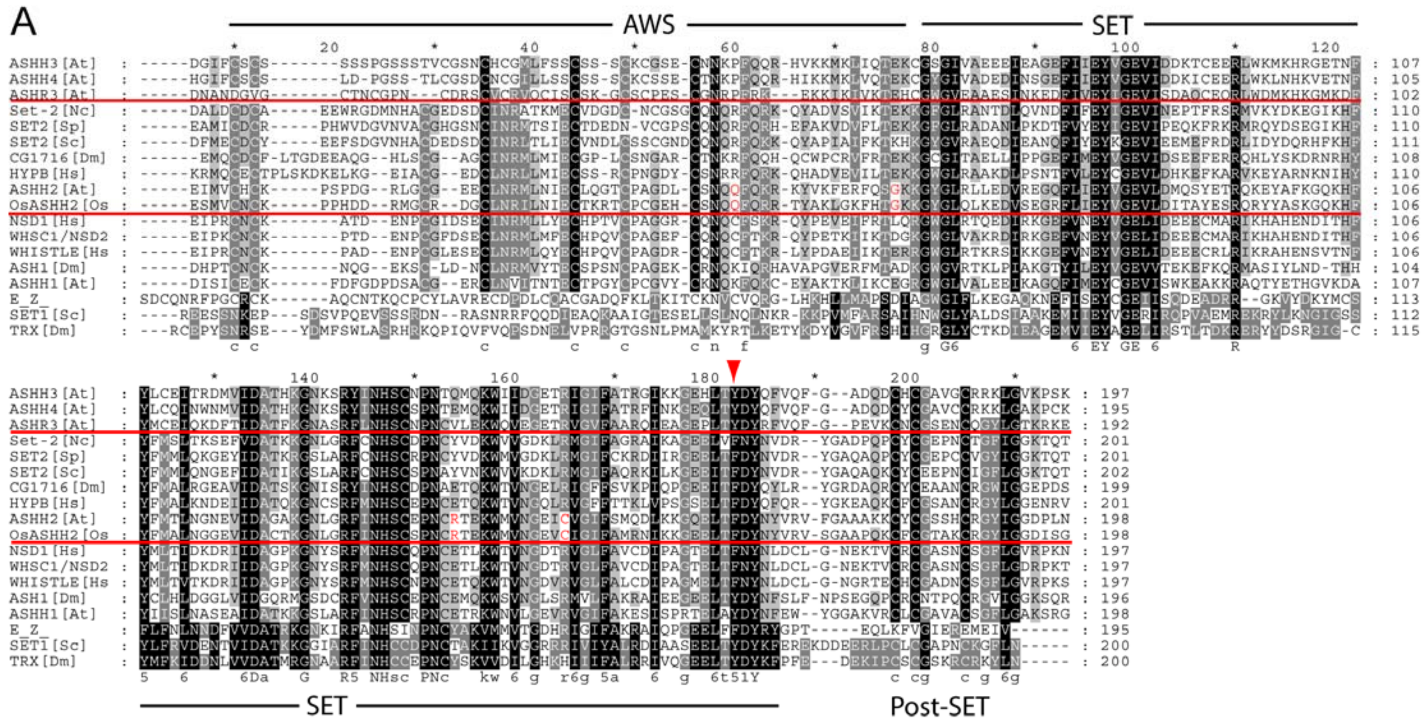

Figure S5. Alignment of the ASHH2 SET domain to SET domains of related proteins. The Associated With SET (AWS) domain, the SET domain and the Post-SET domain of ASHH2 aligned with the same domains of ASH1-group proteins from Arabidopsis [At] and other species ([Nc] *Neurospora crassa*, [Sp] *Saccharomyces pombe*, [Sc] *Saccharomyces cerevisiae*, [Dm] *Drosophila melanogaster*, [Os] *Oryza sativa* (rise), [Hs] human). ASHH2 is most similar to proteins with H3K36me3 activity (between the red lines), however, with a few deviating amino acids also found in the rise homologue (red letters) in conserved regions. The F/Y switch assumed to determine product specificity is indicated with a red arrowhead.
