# Supplementary material for: The ASH1 HOMOLOG 2 (ASHH2) Histone H3 Methyltransferase Is Required for Ovule and Anther Development in Arabidopsis
Source: PLoS One. 2009 Nov 12;4(11):e7817. doi: 10.1371/journal.pone.0007817 (PMC2772814; doi:10.1371/journal.pone.0007817)
Supplement: Figure S6 — Changes in histone tail methylation in the ashh2-1 mutant. (0.09 MB PDF) [file pone.0007817.s006.pdf]

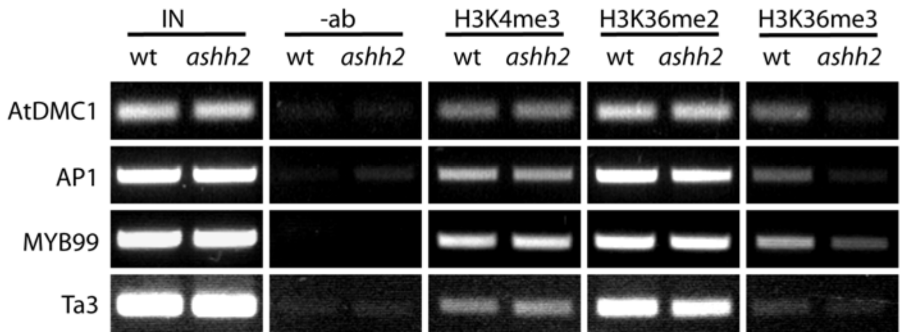

Figure S6. Changes in histone tail methylation in the *ashh2-1* mutant. A second biological replicate of ChIP analyses on the down-regulated genes *AP1*, *AtDMC1* and *MYB99* using antibodies against H3K4me3, H3K36me2 and H3K4me3. *Ta3* was used as a control. IN – input; –ab – without antibodies.
