# Supplementary material for: The ASH1 HOMOLOG 2 (ASHH2) Histone H3 Methyltransferase Is Required for Ovule and Anther Development in Arabidopsis
Source: PLoS One. 2009 Nov 12;4(11):e7817. doi: 10.1371/journal.pone.0007817 (PMC2772814; doi:10.1371/journal.pone.0007817)
Supplement: Table S1 — Frequency of homeotic transformations in different ashh2 alleles. (0.03 MB PDF) [file pone.0007817.s007.pdf]

Table S1. Homeotic transformations in different *ashh2* alleles.

Allele	Number of flowers with transformations	% flowers with transformations	Inflorescences with transformations	% inflorescences with transformation
Col wt	0 of 94	0%	0 of 14	0%
ashh2-1	15 of 217	6.90 %	10 of 65	15.38 %
ashh2-2	28 of 333	8.40 %	18 of 91	19.78 %
ashh2-5	23 of 287	8.01 %	16 of 72	22.22 %
ashh2-6	1 of 70	1.43 %	1 of 6	16.67 %
efs1-1	16 of 262	6.10 %	13 of 54	24.07 %
