# Supplementary material for: The ASH1 HOMOLOG 2 (ASHH2) Histone H3 Methyltransferase Is Required for Ovule and Anther Development in Arabidopsis
Source: PLoS One. 2009 Nov 12;4(11):e7817. doi: 10.1371/journal.pone.0007817 (PMC2772814; doi:10.1371/journal.pone.0007817)
Supplement: Table S2 — Up-regulated genes in ashh2 inflorescences encoding transcription factors and factors involved in development. (0.10 MB PDF) [file pone.0007817.s008.pdf]

Table S2. Up-regulated genes in *ashh2* inflorescences encoding transcription factors and factors involved in development.

AtGID	Name	Reference	Log $_2$-ratio	Fold up
At1g01470	LEA14	Kimura et al., Photochem Photobiol 77: 226-233 (2003)	1.13	2.19
At1g02820	LEA3 familiy protein	-	0.76	1.69
At1g04250	AXR3/IAA17	Ouellet et al., Plant Cell 13: 829–841 (2001)	0.77	1.70
At1g12260	VND4	Kubo et al., Genes Develop 19: 1855–1860 (2005)	0.77	1.70
At1g22590	AGL87/Mγ MADS-box factor	-	2.28	4.86
At1g32870	ANAC 013	Ooka et al., DNA Res 10: 239-247 (2003)	0.92	1.90
At1g52690	LEA protein	Tai et al., Plant Mol Biol 59: 909–925 (2005)	0.74	1.67
At1g52890	ANAC 019	Ooka et al., DNA Res 10: 239-247 (2003)	0.71	1.64
At1g80840	WRKY40	Xu et al., Plant Cell 18:1310-1326 (2006)	0.85	1.80
At2g18550	AtHB21	Henriksson et al., Plant Physiol 139: 509–518 (2005)	0.96	1.95
At2g30250	WRKY25	Andreasson et al., EMBO J 24: 2579-89 (2005)	0.74	1.67
At2g36080	DNA-binding protein	-	0.96	1.95
At2g46680	AtHB7	Soderman et al., Plant J 10: 375-81 (1996)	0.81	1.75
At2g47190	MYB2	Yoo et al., J Biol Chem 280: 3697-3706 (2005)	0.96	1.94
At2g47270	bHLH	Heim et al., Mol Biol Evol 20: 735-747 (2003)	0.91	1.88
At3g01600	ANAC 047	Ooka et al., DNA Res 10: 239-247 (2003)	1.48	2.78
At3g04070	ANAC 045	Ooka et al., DNA Res 10: 239-247 (2003)	0.82	1.76
At3g05800	bHLH	Heim et al., Mol Biol Evol 20: 735-747 (2003)	1.27	2.41
At3g20210	DELTA-VPE	Nakaune et al., Plant Cell 17: 876-887 (2005)	0.73	1.66
At3g23050	AXR2/IAA7	Nakamura et al., Plant J 45: 193–205 (2006)	1.32	2.50
At3g28857	DNA-binding protein	-	0.74	1.67
At3g43160	MEE 38	Pagnussat et al., Development 132: 603-614 (2004)	0.78	1.72
At3g53440	DNA-binding protein	-	0.71	1.63
At3g61630	CRF6	Rashotte et al., PNAS 103:11081-11085 (2006)	0.82	1.77
At4g36740	AtHB40	Henriksson et al., Plant Physiol 139: 509–518 (2005)	0.95	1.94
At5g13330	RAP2.6	Nakano et al., Plant Physiol 140: 411–432 (2006)	0.95	1.93
At5g15160	bHLH	Heim et al., Mol Biol Evol 20: 735-747 (2003)	0.96	1.94
At5g37260	MYB73	Yanhui et al., Plant Mol Biol 60: 107–124 (2006)	0.78	1.72
At5g57520	ZFP2	Tague & Goodman, Plant Mol Biol 28: 267-279 (1995)	1.07	2.10
At5g62430	CDF1	Imaizumi et al., Science 309 :293-297 (2005)	0.79	1.73
