# Supplementary material for: The ASH1 HOMOLOG 2 (ASHH2) Histone H3 Methyltransferase Is Required for Ovule and Anther Development in Arabidopsis
Source: PLoS One. 2009 Nov 12;4(11):e7817. doi: 10.1371/journal.pone.0007817 (PMC2772814; doi:10.1371/journal.pone.0007817)
Supplement: Table S3 — Percentage of pollen showing abnormal exine layers. (0.02 MB PDF) [file pone.0007817.s009.pdf]

Table S3. Percentage of pollen showing abnormal exine layer using SEM.

Mutant line	Abnormal phenotype	Wild type phenotype	N
ashh2-1	26,3 %	73,7 %	38
ashh2-2	79,7 %	20,3 %	74
ashh2-5	96,0%	4,0%	126
ashh2-6	98,1%	1,9%	106
