# Supplementary material for: The ASH1 HOMOLOG 2 (ASHH2) Histone H3 Methyltransferase Is Required for Ovule and Anther Development in Arabidopsis
Source: PLoS One. 2009 Nov 12;4(11):e7817. doi: 10.1371/journal.pone.0007817 (PMC2772814; doi:10.1371/journal.pone.0007817)
Supplement: Table S4 — Genes down-regulated in ashh2 inflorescences and ms1 young buds. (0.13 MB PDF) [file pone.0007817.s010.pdf]

Table S4. Genes down-regulated in *ashh2* inflorescences (>1.6-fold) and *ms1* young buds (> 2-fold).

AtGID	Name/Protein	AtGID	Name/Protein
At1g02790	PGA4 exopolygalacturonase	**At2g47040**	VDG1
At1g02813	expressed protein	**At2g47050**	pectin methylesterase inhibitor
At1g06260	cysteine proteinase	**At3g01270**	pectate lyase
**At1g07340**	ATSTP2	At3g03910	glutamate dehydrogenase
At1g13140	CYP86C3 protein	At3g07850	exopolygalacturonase
At1g13150	CYP86C4 protein	**At3g13400**	SKS13
**At1g15460**	anion exchange family protein	**At3g15900**	expressed protein
**At1g18280**	LTP	*At3g25050*	XTH3
**At1g20120**	GDSL-like Lipase	**At3g26125**	CYP86C2 protein
At1g23240	caleosin-related protein, pollen coat	**At3g28790**	expressed protein
At1g23250	caleosin-related protein, pollen coat	At3g28830	expressed protein
At1g23560	expressed protein	**At3g28980**	expressed protein
At1g23570	expressed protein	At3g51000	epoxide hydrolase
At1g23600	expressed protein	***At3g51590***	LTP12
At1g23670	expressed protein	***At3g52160***	beta-ketoacyl-CoA synthase
At1g23690	expressed protein	At3g52810	ATPAP21
**At1g24400**	AATL2	At3g57620	glyoxal oxidase-related protein
At1g26710	expressed protein	**At3g57690**	AGP23
At1g28375	expressed protein	At3g57810	expressed protein
At1g28430	CYP705A24 protein	**At4g04460**	aspartyl protease
At1g30020	expressed protein	**At4g08670**	LTP
At1g30350	pectate lyase protein	At4g10260	pfkB-type carbohydrate kinase
At1g47980	expressed protein	**At4g13560**	LEA protein
At1g50310	STP9	**At4g14815**	LTP
At1g54860	expressed protein	At4g16160	Tim17/Tim22/Tim23 protein
At1g54870	SDR protein	At4g23660	ATPPT1
**At1g59740**	POT protein	**At4g29250**	Transferase protein
At1g61110	AtNAC025	At4g35770	SEN1
**At1g61566**	RALFL9	**At4g37900**	glycine-rich protein
At1g63060	expressed protein	At5g07410	GRP14, pollen coat
**At1g63180**	UDP-glucose 4-epimerase	At5g07520	GRP18, pollen coat
**At1g67990**	caffeoyl-CoA 3-O-methyltransferase	*At5g07530*	GRP17, pollen coat
At1g68875	expressed protein	At5g07540	GRP16, pollen coat
**At1g71160**	beta-ketoacyl-CoA synthase	**At5g07550**	GRP19, pollen coat
**At1g72280**	AERO 1	**At5g07560**	GRP20, pollen coat
At1g74540	CYP98A8 protein	At5g09550	Rab GDP dissociation inhibitor
**At1g74550**	CYP98A8 protein	***At5g13380***	auxin-responsive GH3 protein
**At1g75910**	EXL4, pollen coat	**At5g14980**	esterase/lipase/thioesterase
At1g75920	EXL5, pollen coat	**At5g20710**	BGAL7
**At1g75930**	EXL6, pollen coat	At5g28470	POT protein
At1g75940	ATA27	At5g37810	major intrinsic family protein
**At1g76470**	cinnamoyl-CoA reductase	**At5g38760**	expressed protein
**At1g80660**	AHA9	*At5g44400*	FAD-binding domain-containing protein
**At2g03740**	LEA protein	**At5g45880**	pollen Ole e 1 allergen
**At2g03850**	LEA protein	At5g46795	expressed protein
At2g07560	AHA6	**At5g47635**	expressed protein
At2g16750	protein kinase	**At5g48210**	expressed protein
At2g18420	gibberellin-responsive protein	**At5g49070**	beta-ketoacyl-CoA synthase
At2g19070	transferase protein	At5g52360	actin-depolymerizing factor
At2g21490	dehydrin	At5g53820	expressed protein
At2g23800	GGPS2	**At5g59845**	gibberellin-regulated protein
At2g31980	cysteine proteinase inhibitor	*At5g60500*	UPP synthetase
At2g32890	expressed protein	At5g61605	expressed protein
At2g41040	methyltransferase-related protein	***At5g62320***	MYB99
**At2g41290**	strictosidine synthase	**At5g65205**	SDR protein
At2g46860	inorganic pyrophosphatase		

Genes in italics induced by MS1 (Ito et al., 2007). Genes in bold also down-regulated by *spl/ems1* (Wijeratne et al., 2007).
