# Supplementary material for: The ASH1 HOMOLOG 2 (ASHH2) Histone H3 Methyltransferase Is Required for Ovule and Anther Development in Arabidopsis
Source: PLoS One. 2009 Nov 12;4(11):e7817. doi: 10.1371/journal.pone.0007817 (PMC2772814; doi:10.1371/journal.pone.0007817)
Supplement: Table S6 — List of primers. (0.13 MB PDF) [file pone.0007817.s012.pdf]

Table S6. List of primers.

Primer name	Application	Oligo sequence	Gene name	AGI code
ASHH2_65460_R2	Genotyping	5'-TTGGGTACACATGGATCAAATAAG-3'	ASHH2	At1g77300
ASHH2_65480_F2	Genotyping	5'-TTGCCTATTTGTTATGTCAGTAATGC-3'	ASHH2	At1g77300
SALK026442_SW_LP	Genotyping	5'-GCTGGGGGTTTATGTAGGAAG-3'	ASHH2	At1g77300
SALK026442_SW_RP	Genotyping	5'-CACTGTCCAGTAAAAGCTGGC-3'	ASHH2	At1g77300
SALK040477_SW_LP	Genotyping	5'-CGTTTTCCTTACAGTCCATCG-3'	ASHH2	At1g77300
SALK040477_SW_RP	Genotyping	5'-GGGGCTGTGTGAGTCTTATTG-3'	ASHH2	At1g77300
ASHH2_SALK_36941_LP	Genotyping	5'-AAGATGATTACCTCATTCCCA-3'	ASHH2	At1g77300
ASHH2_SALK_36941_RP	Genotyping	5'-GAAAAGGAACAGAAGAGCAAG-3'	ASHH2	At1g77300
IPCR15	Genotyping	5'-ACCGTTGCGGTTCTGTCAGTT-3'	-	T-DNA Right Border
J504	Genotyping	5'-GCGTGGACCGCTTGCTGCAACTCTCTCAGG-3'	-	T-DNA Left Border
LBa1	Genotyping	5'-TGGTTCACGTAGTGGGCCATCG-3'	-	T-DNA Left Border
Ashh2_probe_14_LP	RT	5'-CAACAGCAGCCCATCAAAT-3'	ASHH2	At1g77300
Ashh2_probe_14_RP	RT	5'-CTCGTTCAGTTTCCCTTCAAA-3'	ASHH2	At1g77300
PI_probe_7_LP	RT	5'-AAAGAGAATGATAGCTTACAACTGGAG-3'	PI	AT5G20240
PI_probe_7_RP	RT	5'-TCGACAGCCATCAGATTTTTC-3'	PI	AT5G20240
AP1_probe_89_LP	RT, ChIP	5'-ATATGCCTCCCCCTCTGC-3'	AP1	AT1G69120
AP1_probe_89_RP	RT, ChIP	5'-GGATCATCTTCTTGATACAGACCA-3'	AP1	AT1G69120
AP3_probe_119_LP	RT	5'-CGAGCGCAAGTTCAAATCT-3'	AP3	AT3G54340
AP3_probe_119_RP	RT	5'-TTCAGCTCTTAGTTCCAGCTCA-3'	AP3	AT3G54340
AtDMC1_PL_LP	RT, ChIP	5'-CAGGGTGTCAAGCTCTCGAT-3'	AtDMC1	AT3G22880
AtDMC1_PL_RP	RT, ChIP	5'-CTGTGATGGCTGAGGTTTCA-3'	AtDMC1	AT3G22880
MYB99 Int F	ChIP	5'-CAAAACTGGCGGGGCTAAGGAG-3'	MYB99	AT5G62320
MYB99 Int R	ChIP	5'-CTCCACTGCAATCTTCGACCATCTA-3'	MYB99	AT5G62320
Ta3F	ChIP	5'-GAGAGCAAACAGAGTGAGGCTCG-3'	Ta3	Ta3 retrotransposon
Ta3R	ChIP	5'-TTGCTTCAGTTTTGGTAAGCGGA-3'	Ta3	Ta3 retrotransposon
SP/QRT3 269-1860)	RT	5'-CACAGGCCTCAAGTATAACCC-3'	QRT 3	AT4G20050
ASP/QRT3 269-1860	RT	5'-TTAGCTCCATGGTTTCCTCC-3'	QRT 3	AT4G20050
SP/UNE15 31-440	RT	5'-CACAAACTTCAAATCCTCATCATC-3'	UNE15	AT4G13560
ASP/UNE15 31-440	RT	5'-TTTCTCTTGAGCTTGGCCTC-3'	UNE15	AT4G13560
SP/MEE48 591-1588	RT	5'-ATTCTCTCCGGTCGTCGTTTC-3'	MEE48	AT4G14080
ASP/MEE48 591-1588	RT	5'-CGGAAGTAAGGATGAACATTAAGG-3'	MEE48	AT4G14080
MYB99 UPL 1 F	RT	5'-GCTCGCCTTGGCAATAGA-3'	MYB99	AT5G62320
MYB99 UPL1 R	RT	5'-GCTTCCTCTTTATATGAGTGTTCCA-3'	MYB99	AT5G62320
At5g61430 UPL1 F	RT	5'-TGAGTACAGGCTTGAAGGAAAA-3'	NAC5	At5g61430
At5g61430 UPL1 R	RT	5'-CCTGCATATCACCCATTCATT-3'	NAC5	At5g61430
At1g61110 UPL1 F	RT	5'-CAAAGACCAACAATGGAGAGAGT-3'	NAC25	At1g61110
At1g61110 UPL1 R	RT	5'-AGAATTAGCAGATGATTTTGAGAGC-3'	NAC25	At1g61110
ACT2_102 left	RT	5'-CGCTCTTTCTTTCCAAGCTC-3'	ACTIN2	AT3G18780
ACT2_102 right	RT	5'-CCGGTACCATTGTCACACAC-3'	ACTIN2	AT3G18780
2135L	In situ	5'-ATAAAGAGGGTCTGCATCATGG-3'	ASHH2	At1g77300
3115R	In situ	5'-CATAACCCTTCTTACCGGATTG-3'	ASHH2	At1g77300
promGUS_ASHH2_attB1	Cloning	5'-GGGACAAGTTTGTACAAAAAAGCAGGC TTCTCATCTTCAATATGGACGGTGTCC-3'	ASHH2	At1g77300
promGUS_ASHH2_attB2	Cloning	5'-GGGACCACTTTGTACAAGAAAGCTGGGT CCGCCGCAACAGAGACTAAATAA-3'	ASHH2	At1g77300
